# Unraveling the transcriptomic signatures of Parkinson’s disease and major depression using single-cell and bulk data

**DOI:** 10.3389/fnagi.2023.1273855

**Published:** 2023-11-07

**Authors:** Christiana C. Christodoulou, Anna Onisiforou, Panos Zanos, Eleni Zamba Papanicolaou

**Affiliations:** ^1^Neuroepidemiology Department, The Cyprus Institute of Neurology and Genetics, Nicosia, Cyprus; ^2^The Cyprus Institute of Neurology and Genetics Is a Full Member of the European Reference Network-Rare Neurological Diseases (ERN-RND), Tübingen, Germany; ^3^Translational Neuropharmacology Laboratory, Department of Psychology, University of Cyprus, Nicosia, Cyprus

**Keywords:** Parkinson’s disease, major depressive disorder, single-cell RNA, transcriptomics, prefrontal cortex, bulk transcriptomics

## Abstract

**Background:**

Motor symptoms are well-characterized in Parkinson’s disease (PD). However, non-motor symptoms, such as depression, are commonly observed and can appear up to 10 years before motor features, resulting in one-third of individuals being misdiagnosed with a neuropsychiatric disorder. Thus, identifying diagnostic biomarkers is crucial for accurate PD diagnosis during its prodromal or early stages.

**Methods:**

We employed an integrative approach, combining single nucleus RNA and bulk mRNA transcriptomics to perform comparative molecular signatures analysis between PD and major depressive disorder (MDD). We examined 39,834 nuclei from PD (GSE202210) and 32,707 nuclei from MDD (GSE144136) in the dorsolateral prefrontal cortex (dlPFC) of Brodmann area 9. Additionally, we analyzed bulk mRNA peripheral blood samples from PD compared to controls (GSE49126, GSE72267), as well as MDD compared to controls (GSE39653).

**Results:**

Our findings show a higher proportion of astrocytes, and oligodendrocyte cells in the dlPFC of individuals with PD vs. MDD. The excitatory to inhibitory neurons (E/I) ratio analysis indicates that MDD has a ratio close to normal 80/20, while PD has a ratio of 62/38, indicating increased inhibition in the dlPFC. Microglia displayed the most pronounced differences in gene expression profiles between the two conditions. In PD, microglia display a pro-inflammatory phenotype, while in MDD, they regulate synaptic transmission through oligodendrocyte-microglia crosstalk. Analysis of bulk mRNA blood samples revealed that the *COL5A*, *MID1*, *ZNF148*, and *CD22* genes were highly expressed in PD, whereas the *DENR* and *RNU1G2* genes were highly expressed in MDD. *CD22* is involved in B-cell activation and the negative regulation of B-cell receptor signaling. Additionally, *CD86*, which provides co-stimulatory signals for T-cell activation and survival, was found to be a commonly differentially expressed gene in both conditions. Pathway analysis revealed several immune-related pathways common in both conditions, including the complement and coagulation cascade, and B-cell receptor signaling.

**Discussion:**

This study demonstrates that bulk peripheral immune cells play a role in both conditions, but neuroinflammation in the dlPFC specifically manifests in PD as evidenced by the analysis of single nucleus dlPFC datasets. Integrating these two omics levels offers a better understanding of the shared and distinct molecular pathophysiology of PD and MDD in both the periphery and the brain. These findings could lead to potential diagnostic biomarkers, improving accuracy and guiding pharmacological treatments.

## 1. Introduction

Parkinson’s disease (PD) is the second most common neurodegenerative disease (ND), affecting approximately 2–3% of the population aged 65 years and older, with increasing incidence worldwide (Poewe et al., 2017; Ntetsika et al., 2021). PD is a chronic movement disorder of the central nervous system (CNS) (Kalia and Lang, 2015; Poewe et al., 2017), characterized by early motor symptoms such as tremor, difficulty walking, rigidity, and slowness of movement.

In addition to motor symptoms, PD presents with various non-motor clinical features that significantly contribute to the overall disease burden. These include autonomic dysfunction, cognitive impairment, depression, constipation, hyposmia (smell impairment), and insomnia (Kalia and Lang, 2015; Poewe et al., 2017). Some non-motor symptoms of PD, such as constipation and neuropsychiatric symptoms (NPS) like depression and anxiety, may appear up to 10 years before the onset of motor symptoms (Abbott et al., 2001; Jacob et al., 2010; Schrag et al., 2015). The presence of these prodromal non-motor symptoms often leads to low diagnostic accuracy during the early stages of PD, with up to 50% of cases being misdiagnosed (Adler et al., 2014). Depression is a prominent non-motor symptom of PD, affecting approximately 40% of patients (Schrag et al., 2015). Furthermore, around one-third of individuals with PD are misdiagnosed with neuropsychiatric disorders (NPDs) during the prodromal or early stages, leading to inappropriate treatment (Woolley et al., 2011). Thus, it is crucial to identify diagnostic biomarkers that can differentiate between depression arising from PD pathophysiology and other factors, such as trauma.

The main pathological hallmark of PD is neuronal loss in the substantia nigra pars compacta, resulting in striatal dopamine deficiency and the widespread accumulation of aggregated α-synuclein (Poewe et al., 2017). During the early stages of the disease, the loss of dopaminergic neurons is primarily constrained to the Ventrolateral Substantia Nigra, while other midbrain dopaminergic neurons remain relatively unaffected. However, as the disease progresses, neuronal loss becomes more widespread (Poewe et al., 2017).

Previous studies have investigated the role and involvement of the prefrontal cortex (PFC) in the pathogenesis of both PD and Major Depressive Disorder (MDD) (Brück et al., 2004; Zhou et al., 2019). The PFC seems to be the main brain region involved in the emergence of PD cognitive symptoms, including executive dysfunction and thought disorders, possibly due to altered prefrontal dopamine signaling (Narayanan et al., 2013). Early-stage PD patients exhibit higher activation of the dorsolateral PFC (dlPFC) during normal walking compared to controls, which was suggested to be a compensatory mechanism for poor executive functioning (Ranchet et al., 2020). The PFC has also emerged as one of the regions consistently impaired in MDD, both in those with current MDD and those with an increased vulnerability to MDD (Pizzagalli and Roberts, 2022). PFC dysfunction has been associated with disordered thought and depression in NDs, including PD. However, further research is needed to gain insight into the pathophysiological explanation of cognitive impairment in PD and the potential association of behavioral impairment with prefrontal dysfunction (Narayanan et al., 2013; Pizzagalli and Roberts, 2022).

The rapid advancement of next-generation sequencing (NGS) technologies in recent years has provided valuable insights into complex biological diseases, ranging from cancer and NDs to diverse microbial communities (Hwang et al., 2018). NGS-based technologies, including transcriptomics, are now increasingly focused on characterizing individual cells (Hwang et al., 2018). These single-cell analyses allow researchers to uncover new and potentially unexpected biological discoveries, which may not be evident through traditional profiling approaches that assess bulk populations (Hwang et al., 2018). For example, single-cell RNA sequencing (scRNA-seq) can reveal rare and complex cell populations, unveil regulatory relationships between genes, and track the trajectories of distinct cell lineages during development (Hwang et al., 2018).

In this study, we compared the molecular signatures of PD and MDD by leveraging publicly available transcriptomic datasets. We integrated post-mortem dlPFC Brodman area 9 (BA9) single-nucleus RNA sequencing (snRNA-seq) samples and peripheral blood bulk mRNA samples obtained from patients with PD and MDD. By analyzing PD and MDD at the single-cell resolution in the dlPFC, we characterized the relative proportion of specific cell types associated with each condition. Additionally, we identified differences in their gene expression patterns and pinpointed the functional biological processes that relate to the differentially expressed genes (DEGs) of PD and MDD in each cell type. At the bulk mRNA level, comparing blood samples between PD and MDD allowed us to characterize the similarities and differences in their gene expression patterns in the periphery. Furthermore, we identified the biological processes (BP), cellular components (CC), and molecular functions (MF) associated with these expression patterns. This integrative approach enabled us to combine high- and low-resolution data from different tissues (brain and periphery) and unravel the shared and divergent molecular signatures of PD and MDD with greater accuracy. As a result, we uncovered novel insights that can potentially contribute to the discovery of diagnostic biomarkers.

## 2. Materials and methods

**Figure 1:**
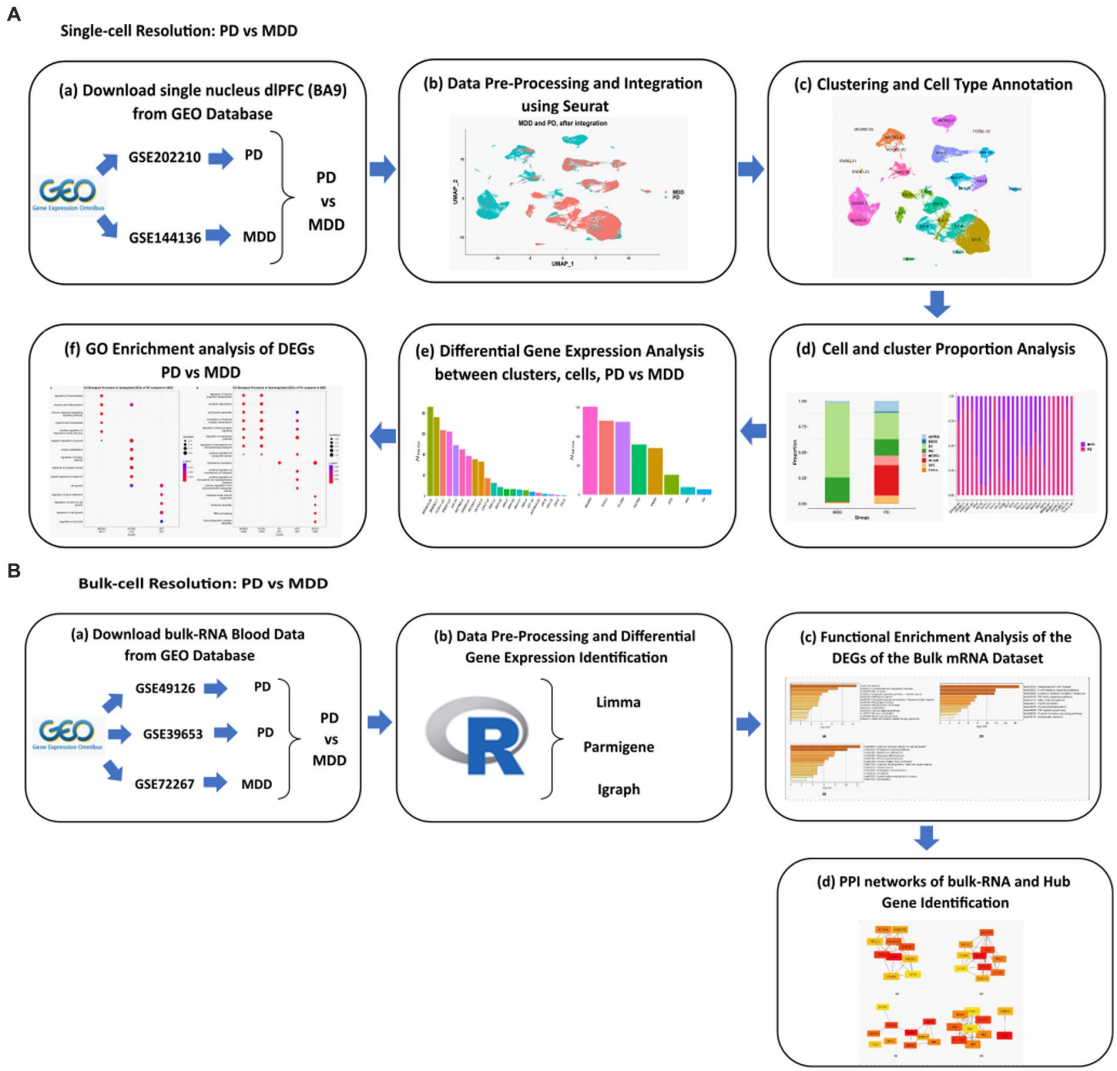
Schematic representation of the workflows applied for the analysis of the **(A)** sn-RNA seq dlPFC datasets and **(B)** bulk mRNA peripheral blood mononuclear cells datasets of PD vs. MDD.

The workflow implemented in this study is illustrated in Figure 1.

### 2.1. GEO dataset information

We extensively searched the Gene Expression Omnibus (GEO) database, a resource provided by the National Center for Biotechnology Information (NCBI) (Edgar et al., 2002), for high-throughput gene expression and other functional genomics datasets related to PD and MDD (Edgar et al., 2002). Our search aimed to identify scRNA-seq and transcriptomics data that met the following criteria: (i) scRNA-seq datasets for PD and MDD containing the dlPFC region BA9; (ii) transcriptomics datasets for PD and MDD in peripheral blood mononuclear cells (PBMC). Based on these criteria, we identified the single-nucleus transcriptomic datasets with accession numbers GSE202210 (Zhu et al., 2022) and GSE144136 (Nagy et al., 2020), as well as the PBMC bulk transcriptomic datasets with accession numbers GSE49126 (Mutez et al., 2014), GSE72267 (Calligaris et al., 2015) and GSE39653 (Savitz et al., 2013). For detailed information regarding the collection and sequencing of the post-mortem brain and peripheral blood samples included in these datasets, please refer to the respective papers associated with each dataset.

#### 2.1.1. Single-nucleus RNA dlPFC datasets

The GSE202210 dataset contains tissue samples from the dlPFC obtained from three female and three male patients with PD, with ages ranging from 72 to 96, along with six non-diseased gender/age-matched controls. The six PD patients had tau Braak stage ranging from levels I-III. On the other hand, the GSE144136 dataset consists of dlPFC tissue samples obtained from 17 male patients with MDD, along with 17 non-diseased gender/age-matched controls (Nagy et al., 2020). The cause of death of the 17 MDD cases was suicide, and their mean average age was 41.06 ± 4.66 years. For the purpose of this study, only the sn-RNA data from the six PD patients and 17 MDD patients were used for analysis, while the control samples from both datasets were excluded. For a detailed description of the datasets, please refer to Table 1. The count matrix, barcodes, and features files for both datasets were downloaded from the GEO database and utilized for the analysis.

**Table 1:** Detailed information of the sn-RNA transcriptomics datasets of PD and MDD analyzed in this study.

Accession ID	Platform	Platform type	Sample size (case/control)	Sample type
GSE202210	GPL24676	Illumina NovaSeq 6000	6 PD	Post-mortem brain tissue (BA9 area dlPFC)
GSE144136	GPL20301	Illumina HiSeq 4000	17 MDD	Post-mortem brain tissue (BA9 area dlPFC)

#### 2.1.2. Bulk-mRNA peripheral blood datasets

The GSE49126 dataset consisted of 9 individuals heterozygous for LRRK2 G2019S mutation (mean age: 66.2 ± 17; 4 males and 5 females) and 20 individuals with sporadic PD (mean age: 62.3 ± 8; 9 males and 11 females). Twenty controls (20 males, 20 females, mean age 52.9 ± 19) were recruited from volunteers with no history of personal or familial neurological disorders (Mutez et al., 2014). The GSE72267 dataset consists of 39 PD patients and 20 controls with mean ages of 68.8 years and 60.3 years, respectively. Lastly, the GSE39653 dataset consisted of 8 individuals with bipolar disorder (BD) (4 BD type 1 and 4 BD type 2), 21 individuals with MDD with a mean age of 35 ± 10, and 24 healthy controls with a mean age of 34 ± 12 (Savitz et al., 2013). Regarding GSE39653 (Savitz et al., 2013), only MDD patients and controls were used for further analysis, while BD type 1 and type 2 samples were excluded. Table 2 provides a detailed description of the included datasets.

**Table 2:** Detailed information of the bulk mRNA peripheral blood datasets of PD and MDD analyzed in this study.

Accession ID	Platform	Platform type	Sample size (case/control)	Sample type
GSE49126	GPL4133	Agilent 014850 Whole Genome Microarray 4x44K G4112F	30 PD/20 Controls	Peripheral blood mononuclear cells
GSE72267	GPL571	Affymetrix Human Genome U133A 2.0 Array	39 PD/20 Controls	Peripheral blood mononuclear cells
GSE39653	GPL10558	Illumina HumanHT-12 V4.0 expression beadchip	21 MDD/24 Controls	Peripheral blood mononuclear cells

### 2.2. Analysis of the single-nucleus post-mortem brain samples form PD and MDD patients

#### 2.2.1. Pre-processing and integration analysis of the single-nucleus datasets

Starting from the unique molecular identifier (UMI) count matrices of the two dlPFC snRNA-seq datasets of 6 patients with PD (GSE202210) and 17 patients (6 Batches) with MDD (GSE144136) without control cases, we used the Seurat (version 4.0.2) single-cell analysis R package (Hao et al., 2021) to perform quality control (QC) on both datasets. Low-quality nuclei with either less than 1,000 features (genes) or more than 6,000 features were removed from the MDD dataset. For the PD dataset, low-quality nuclei with either less than 200 features or more than 6,000 features and a mitochondrial percentage greater than 5 were removed. The MDD dataset did not contain any mitochondrial genes as they were removed by the authors (Nagy et al., 2020) who provided the GSE144136 dataset. To perform the integration between the two datasets, we limited the comparison only to their common genes (features), resulting in individual matrices limited to 20,093 common genes (features) profiled in 39,834 and 32,707 nuclei in PD and MDD, respectively. Before performing the integration, each UMI dataset was normalized, and the 2000 most variable features across all nuclei in each sample were identified using Seurat. Then, the PD and MDD experimental conditions were normalized (using the Seurat function NormalizeData) and integrated using the Seurat data integration workflow.

#### 2.2.2. Clustering and cell type annotation

After integrating the MDD and PD datasets, the integrated data were scaled using the ScaleData function of Seurat, and dimensionality reduction using principal components analysis (PCA) was performed on the first 50 PCs. The PC elbow plot, which visualizes the standard deviation for each PC (Supplementary File 1 Figure S1), was used to determine the number of PCs to be used for further analysis. Based on the elbow plot, the first 30 PCs were selected for Uniform Manifold Approximation and Projection for Dimension Reduction (UMAP) and for the FindNeighbors function (Seurat). The FindClusters function (Seurat) was then applied at a resolution of 0.2, resulting in the identification of 26 clusters.

The annotation of the 26 clusters was performed by manually annotating the clusters, utilizing well-established cell markers (Zhang et al., 2019). Additionally, this annotation was guided by the cell markers used in the annotations of the original studies that provided the two datasets (Nagy et al., 2020; Zhu et al., 2022). To annotate these 26 clusters, we made use of specific markers for each cell type. These markers included: astrocytes (*GFAP, AQP4*, and *SLC1A2*), T cells (*SKAP1* and *IL7R*), oligodendrocytes (*PLP1, MOG, MOBP*, and *MBP*), macrophage/microglia (*CSF1R, CD74*, and *PYRY12*), endothelial (*FLT1*, *EBF1*, and *CLDN5*), inhibitory neuron (*GAD1* and *GAD2*), oligodendrocyte precursor cell (OPC) (*CSPG4* and *PDGFRA*), and excitatory neuron (*SATB2* and *SLC17A7*).

#### 2.2.3. Differential gene expression analysis between the clusters, the cells and PD versus MDD

The FindAllMarkers function (Seurat) was used to identify differentially expressed genes (DEGs) that exhibit statistically significant difference in their expression between the clusters and between the cells. DEGs having an average log2FC > 2.0 and adjusted value of *p* < 0.05 were considered as statistically significant. The min.pct and logFC threshold parameters were both set at 0.25, and the Wilcoxon Rank Sum test was used.

Additionally, to gain insight on the average gene expression patterns between PD and MDD we first used the AverageExpression function (Seurat) to identify the average expression level of genes across all samples in microglia cells, astrocytes, excitatory neurons and inhibitory neurons in each condition. Subsequently, the difference between the average gene expression of PD from MDD was found for each of the four cell types. Scatter plots were then used to visualize the gene expression differences between PD vs. MDD, for the four cell types, with the top 10 upregulated and top 10 downregulated genes between the two conditions highlighted on each scatter plot.

Furthermore, we conducted differential gene expression analysis using the normalized RNA data to identify statistically significant genes that are upregulated (average log2FC > 1, adj. value of *p* < 0.05) and downregulated (average log2FC < −1, adj. value of *p* < 0.05) in the cells of PD compared to MDD. The analysis was performed using the FindMarkers function (Seurat), selecting the default parameters and utilizing the Wilcoxon Rank Sum test. T cells were excluded from this segment of the analysis as they were exclusively present in PD samples and absent in MDD samples. These DEGs were subsequently subjected to enrichment analysis.

#### 2.2.4. Reconstruction and analysis of microglia protein–protein interaction networks of the DEGs in PD compared to MDD

The upregulated and downregulated DEGs identified when comparing PD and MDD microglia cells were utilized as input in STRING plug-in of Cytoscape (Doncheva et al., 2019) to create microglia cell-specific PPI networks. Specifically, we constructed two separate networks for the upregulated and downregulated DEGs. Following the construction of the PPI networks, the cytoHubba (Chin et al., 2014) plugin of Cystoscope was used to perform topological analysis on each of these constructed PPI networks. The confidence cut-off score for the PPIs was set at 0.7. The primary objective of this analysis was to pinpoint DEGs that exhibit high connectivity and centrality within PD microglia cells relative to MDD, and vice versa. To achieve this goal, we employed two topological measures: degree centrality and closeness centrality. These measures helped us identify high centrality DEGs within the microglia PPI networks that play significant roles in the specific molecular interactions associated with PD compared to MDD.

#### 2.2.5. Gene ontology enrichment analysis of the DEGs from each cell type

Enrichment analysis was performed using the clusterProfiler package (Wu et al., 2021) in R. For this analysis, we utilized the up- and down-regulated DEGs, identified for each cell when comparing PD condition to MDD condition, specifically focusing on genes with an adjusted value of *p* < 0.05. These genes were used as input in the enrichGO function of clusterProfiler to identify GO biological processes (GO-BP) associated with the upregulated and downregulated genes. The enrichGO function was configured with the following parameters: BP ontology, *p* adjusted method Benjamini & Hochberg (BH), a value of *p* cut-off set at 0.01, and a *q*-value cut-off set at 0.05. The default values were used for all other parameters. Redundant terms were removed from the obtained results using the simplify() method of clusterProfiler.

### 2.3. Analysis of the microarray peripheral blood samples from PD and MDD patients

#### 2.3.1. Data processing and differential gene expression identification

The linear models for microarray data (Limma), an R package that permits the identification of DEGs from high-throughput techniques such as microarray experiments (Ritchie et al., 2015), was used to identify the DEGs for the GSE49126 dataset (PD vs. controls), GSE72267 (PD vs. controls), and GSE39653 (MDD vs. controls) (Ritchie et al., 2015). The GSE49126 and GSE72267 datasets were normalized and log_2_ transformed. Following the Limma analysis, a total of 400 DEGs (top 200 upregulated and top 200 downregulated DEGs) with an adjusted value of *p* < 0.05 were selected for each PD and MDD dataset for further analysis.

The intensity matrix and top IDs for each of the datasets were input into the Parmigene R-package, which performs parallel estimation of mutual information based on estimates from the k-nearest neighbor’s distances and uses algorithms to reconstruct gene regulatory networks (Sales and Romualdi, 2011). The output obtained is a clr file for PD patients vs. controls and MDD patients vs. controls. The R package Igraph (Aittokallio and Schwikowski, 2006) was then used to obtain the edge lists for each dataset. However, to avoid noise within our gene co-expression networks and gain meaningful biological information from each of the networks, a further cutoff threshold of the log function [log(weight)]; was applied to the final edge list for all three datasets. Therefore, only genes with weights of 1 and above were used as inputs in Cytoscape (Shannon et al., 2003) for the construction of PD and MDD gene co-expression networks.

### 2.4. PPI networks of bulk-RNA datasets and hub gene identification

The STRING plug-in of Cytoscape (Doncheva et al., 2019) was used to obtain the PPIs of the PD and MDD gene co-expression networks. The constructed PPI networks were analyzed using the cytoHubba (Chin et al., 2014) plug-in. In addition, cytoHubba was utilized to rank the shared common gene/s obtained using the Venny tool^1^ from all three datasets. CytoHubba ranks the nodes within the biological network using different topological measures, including (i) degree, (ii) closeness, (iii) betweenness, and (iv) maximal clique centrality (MCC) measures. For further information regarding each approach, please refer to the cytoHubba paper (Chin et al., 2014).

### 2.5. Functional enrichment analysis of the DEGs of the bulk mRNA datasets

The top 200 over- and under-expressed genes identified for each dataset were used as inputs in Metascape, an open-source web-based tool that performs enrichment analysis for a variety of organisms (Zhou et al., 2019). In Metascape, terms with a value of *p* < 0.05, a minimum count of 3, and an enrichment factor of >1.5 (the ratio between the observed count and the counts expected by chance) are grouped into clusters based on their membership similarity. *p*-values are calculated based on the cumulative hypergeometric distribution, and *q*-values are based on the Benjamini-Hochberg method for multiple testing. Kappa Scores are used as the similarity metric to perform hierarchical clustering on the enriched terms obtained, and sub-trees with a similarity of >0.3 are clustered together. Only the most statistically significant terms are chosen to be represented within the cluster (Zhou et al., 2019). Enrichment analysis was performed by selecting the *Homo Sapiens* organism using the Kyoto Encyclopedia of Genes and Genomes (KEGG) library and the GO libraries of BP (Zhou et al., 2019), Cellular Components (GO-CC) and Molecular Function (GO-MF) to identify statistically significant biological pathways and processes with a value of *p* < 0.05. In addition, the shared, common, and distinct KEGG pathways and GO terms between the three bulk transcriptomic datasets were identified using the Venny tool (footnote 1).

## 3. Results

### 3.1. Single-nucleus comparative transcriptomic profiling of post-mortem brain tissue samples of PD and MDD

**Figure 2:**
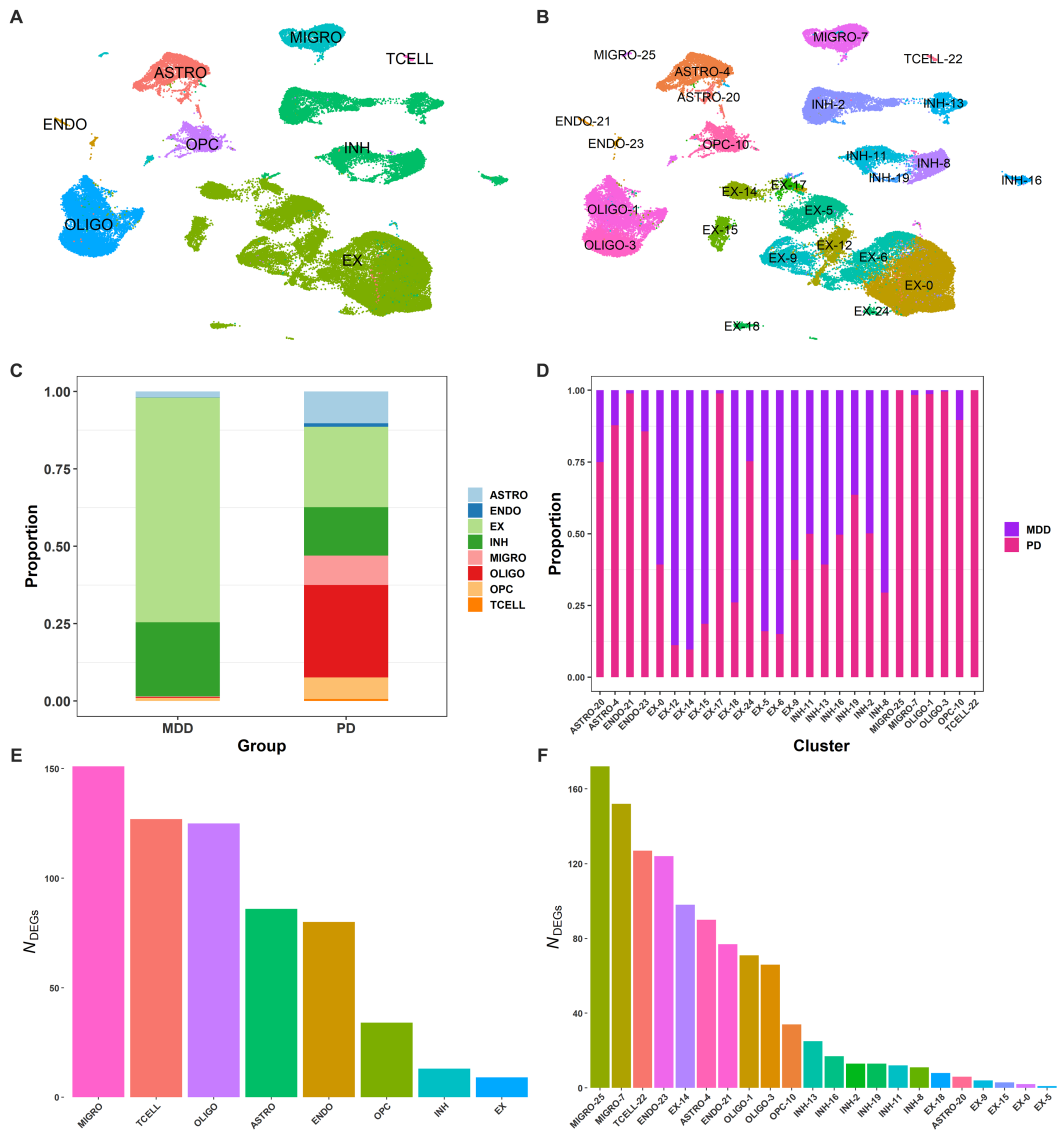
Single-nucleus of dlPFC samples from PD and MDD. **(A)** UMAP plot of the 72,541 brain nuclei post-filtering from MDD and PD, colored by cell type: endothelial (ENDO), microglia (MIGRO), excitatory neurons (EX), inhibitory neurons (INH), oligodendrocytes (OLIGO), brain T cells (TCELL), astrocytes (ASTRO), and oligodendrocyte precursor cells (OPCs). **(B)** UMAP plot of the 26 annotated clusters, indicating the associated cell type for each cluster. **(C)** Proportion of brain nuclei found for each cell type in the MDD and PD conditions. **(D)** Proportion of brain nuclei found for each cluster in the MDD and PD conditions. **(E)** DEG markers (average log2FC >2.0 and adjusted value of *p* < 0.05) found between the 8 cell types. **(F)** DEG markers (average log2FC >2.0 and adjusted value of *p* < 0.05) found between the 26 clusters.

Using publicly available sn-RNA seq data obtained from the dlPFC (BA9) of post-mortem brain tissues from 17 patients with MDD (GSE144136) and 6 patients with PD (GSE202210), we compared their transcriptomic signatures to identify commonalities and differences between the two conditions. By using the common genes between the two datasets, we integrated the two conditions (Butler et al., 2018) and performed quality control. We then clustered the 72,541 post-filtering brain nuclei (MDD and PD; Supplementary File 1 Figure S2) using the first 30 PCs (see Methods 2.2.2), which led to the identification of 26 distinct clusters. By using known cell markers, we annotated the 26 clusters and identified eight cell types: astrocytes with two clusters (ASTRO-4, ASTRO-20), oligodendrocytes with two clusters (OLIGO-1, OLIGO-3), microglia with two clusters (MIGRO-25, MIGRO-7), inhibitory neurons with six clusters (INH-2, INH-13, INH-11, INH-19, INH-16, INH-8), excitatory neurons with ten clusters (EX-14, EX-17, EX-5, EX-15, EX-9, EX-12, EX-6, EX-0, EX-18, EX-24), endothelial with two clusters (ENDO-21, ENDO-23), T cells (TCELL-22), and OPC (OPC-10) (Figures 2A,B; Supplementary File 1 Table S1; Supplementary File 1 Figure S3).

Interestingly, T cells were only identified in PD but not in MDD, with all other seven cell types identified in both conditions (Figure 2C). However, the relative proportion of the seven cell types was different between the two conditions, with PD having significantly more microglia than MDD. This, in combination with the detection of T cells in PD, suggests the presence of neuroinflammation in the dlPFC (BA9) of PD. Additionally, there were more astrocytes in PD, indicating the presence of astrogliosis, as well as significantly more oligodendrocytes and OPCs compared to the MDD condition. Furthermore, the relative proportion of excitatory to inhibitory neurons (E/I ratio) in MDD was 75/25, while in PD it was 62/38. Regarding the clusters of cell types, microglia cluster MIGRO-25 was only found in the PD condition, while clusters ENDO-21 and EX-17 of endothelial and excitatory neurons, respectively, had fewer nuclei in MDD compared to PD (Figure 2D). Moreover, some excitatory neuron clusters showed a higher proportion in the MDD condition than PD, including EX-12, EX-15, EX-5, and EX-6.

We then identified the DEGs between the 26 clusters and between the 8 cell types to determine cluster- and cell-type-specific marker patterns, respectively. DEGs with an average log2FC >2.0 and adjusted value of *p* < 0.05 were considered statistically significant, resulting in a total of 625 DEGs between the eight cell types (Figure 2E) and a total of 1,126 DEGs between the 26 clusters (Figure 2F; Supplementary File 2 Tables S1, S2). The top 3 enriched markers for each cell type were also identified, including *PLP1*, *RNF220*, and *ST18* for oligodendrocytes; *SLC1A2*, *ADGRV1*, and *GPC5* for astrocytes; *LHFPL3*, *VCAN*, and *TNR* for OPCs; *GRIK1*, *ERBB4*, and *ZNF385D* for inhibitory neurons; *RALYL*, *KCNIP4*, and *CHN1* for excitatory neurons; *CEMIP*, *FLT1*, and *ADAMTS9* for endothelial cells; *PTPRC*, *ETS1*, and *PRKCH* for T cells; and *SPP1*, *RUNX1*, and *DOCK8* for microglia.

#### 3.1.1. Differences in the gene expression profile of PD compared to MDD

We employed two distinct approaches to compare the gene expression profiles between PD and MDD cells. Firstly, we examined differences in the average gene expression patterns of PD compared to MDD in microglia cells, astrocytes, excitatory neurons and inhibitory neurons. This initial analysis aimed to broadly identify genes that deviate significantly between the two conditions. Secondly, we conducted a more rigorous analysis to identify statistically significant DEGs that were either upregulated or downregulated in PD compared to all MDD cells. Subsequently, we investigated the functional implications of these identified DEGs in each cell type. To achieve this, we performed GO enrichment analysis to uncover the biological functions associated with these DEGs.

##### 3.1.1.1. Average gene expression

**Figure 3:**
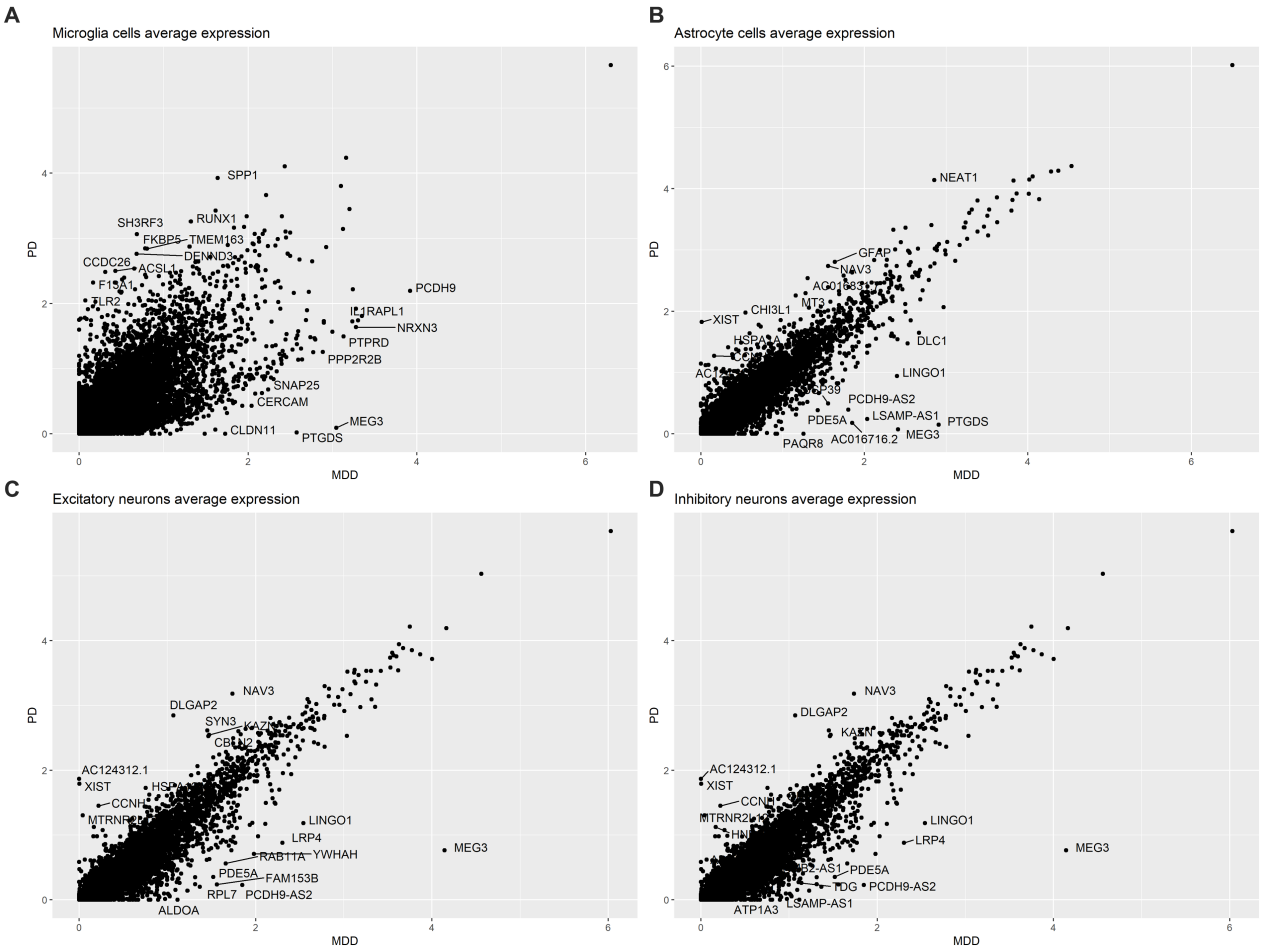
Comparison of the average gene expression profile of PD compared to MDD in: **(A)** microglia cells, **(B)** astrocytes, **(C)** excitatory neurons and **(D)** inhibitory neurons.

The analysis of the average gene expression profiles in microglia cells, astrocytes, excitatory neurons and inhibitory neurons between PD and MDD allowed to identify the top 10 upregulated and top 10 downregulated genes that exhibited significant differences in their average gene expression levels between the two conditions within these cell types. Specifically, our findings reveal that PD microglia cells exhibit higher average expression levels of the *SPP1*, *TLR2* and *CCDC26* genes compared to MDD microglia. On the other hand, MDD microglia exhibit higher average expression levels of the *IL1RAPL1*, *NRXN3* and *SNAP25* genes (Figure 3A). Increased expression of *TLR2* plays a pivotal role in the underlying pathological mechanism contributing to chronic neuroinflammation in PD. When exposed to α-synuclein, microglia become activated, adopting a pro-inflammatory *TLR2* phenotype, which facilitates neurodegeneration (Kim et al., 2013). Additionally, PD astrocytes exhibit higher average expression levels of the glial fibrillary acidic protein (*GFAP*) gene compared to MDD astrocytes (Figure 3B). Elevated *GFAP* expression is a hallmark of astrogliosis, a condition that is characterized by abnormal proliferation and activation of astrocytes that leads to the release various neurotoxic substances in response to nearby neuronal damage (Eng and Ghirnikarz, 1994). The presence of these reactive astrocytes contributes to neuroinflammation, which is a key pathological characteristic of PD (Booth et al., 2017). In contrast to PD, MDD astrocytes exhibit higher expression levels of the Ubiquitin specific protease 39 (*USP39*) gene, which has oncogenic effects and plays a role in various cancers, including promoting glioma progression (Ding et al., 2019; Xiao et al., 2022). Interestingly, patients with glioma have a higher risk of developing depression (Hu et al., 2022), suggesting that *USP39* may serve as a potential bridge between the two conditions. Both excitatory (Figure 3C) and inhibitory neurons (Figure 3D) in PD exhibited similar patterns of higher average expression levels of the *NAV3, DLGAP2, KAZN, MTRNR2L12, CCNH* and *XIST* genes compared to MDD excitatory and inhibitory neurons, which both display higher average expression of the *MEG3, LRP4, LINGO1* and *PDE5A* genes.

##### 3.1.1.2. DEGs analysis and biological functions

Furthermore, a more in-depth analysis analysis of the gene expression profiles across all PD cells compared to MDD was conducted. This involved employing differential gene expression analysis to identify statistically significant DEGs that distinguish the two conditions within each cell type. The differential expression analysis revealed a total of 466 upregulated DEGs (average log2FC >1, adj. value of *p* < 0.05) and 954 downregulated DEGs (average log2FC < −1, adj. value of *p* < 0.05) in the seven PD dlPFC cells when compared to MDD cells (see Supplementary File 2 Tables S3, S4). T cells were excluded from this part of the analysis as they were exclusively found in PD samples and absent in MDD samples. Among the PD dlPFC cells, microglia exhibited the highest number of DEGs (235 upregulated, 371 downregulated), while inhibitory neurons had the fewest number of DEGs (23 upregulated, 49 downregulated) (Table 3). Furthermore, endothelial cells showed only 3 upregulated DEGs and 202 downregulated DEGs in PD compared to MDD.

**Table 3:** Number of upregulated and downregulated DEGs and enriched GO BP found for each cell type in PD compared to MDD.

	Upregulated DEGs	Downregulated DEGs	Upregulated GO BP	Downregulated GO BP
MIGRO	235	371	92	81
OLIGO	81	150	0	64
EX	24	62	0	1
INH	23	49	0	0
ASTRO	48	29	17	0
OPC	52	81	7	15
ENDO	3	202	0	12

**Figure 4:**
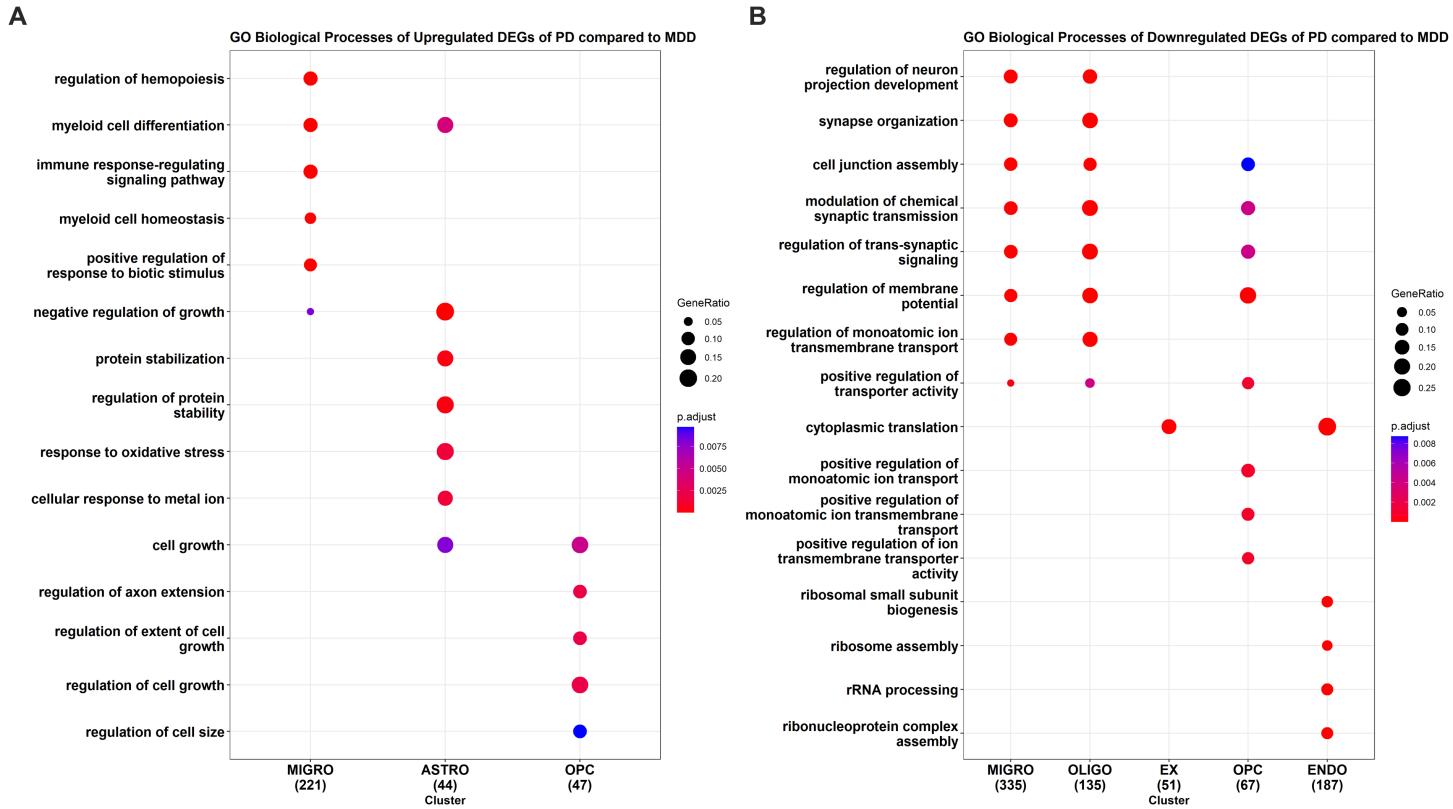
GO enrichment analysis results of the **(A)** upregulated and **(B)** downregulated DEGs of PD compared to MDD for each cell type.

We also conducted GO enrichment analysis to determine the biological processes associated with the upregulated DEGs (Figure 4A) and downregulated DEGs (Figure 4B) in PD cells relative to MDD cells. Our analysis identified a total of 173 downregulated GO BP (see Supplementary File 2 Table S5) and 116 upregulated GO BP (see Supplementary File 2 Table S6) associated with the PD cells relative to MDD. PD microglia showed significant upregulation in biological processes related to hemopoiesis, including myeloid cell differentiation and myeloid cell homeostasis. Additionally, the immune response-regulating signalling pathway, involved in immune response regulation, was also upregulated in microglia. Astrocytes exhibited upregulation in GO terms associated with protein stabilization, regulation of protein stability, negative regulation of growth, myeloid cell differentiation, cellular response to metal ion, and response to oxidative stress. For OPC, the upregulated GO terms were related to cell growth regulation, including regulation of extent of cell growth, regulation of cell growth, and regulation of axon extension. No significant enriched upregulated terms were found for the remaining cell types.

The top downregulated GO terms of PD microglia, compared to MDD microglia, are associated with the regulation of neuron projection development, synapse organization, cell junction assembly, synaptic transmission, and trans-synaptic signalling. Interestingly, these downregulated terms are also observed in oligodendrocyte cells. In excitatory neurons, only the term “cytoplasmic translation” is downregulated, which is also found in endothelial cells. Similarly, like microglia and oligodendrocytes, downregulated terms in PD OPC relate to the regulation of synaptic transmission and trans-synaptic signalling. In addition, downregulated terms in PD OPC include those involved in the positive regulation of monoatomic ion transport. In PD endothelial cells, the top downregulated GO terms are associated with ribonucleoprotein assembly, including ribosome assembly, rRNA processing, and ribosomal small subunit biogenesis. No significant downregulated enriched terms were found in inhibitory neurons and astrocytes of PD cells compared to MDD cells.

##### 3.1.1.3. High centrality DEGs in PD microglia compared to MDD

To identify key DEGs playing significant roles in the biological processes of microglia cells in PD compared to MDD, we constructed microglia cell-specific PPI networks using the upregulated and downregulated DEGs identified in the comparison between PD and MDD (see Table 3). Through topological analysis, we pinpointed the top 5 upregulated DEGs exhibiting the highest degree centrality and closeness centrality within each of the microglia PPI networks.

In the microglia PPI network created with the upregulated DEGs in PD, the DEGs with the highest centrality scores included *STAT3*, *LYN*, *SYK*, and *GRB2*. These genes demonstrated both high degree and closeness centrality. Additionally, *HCK* ranked among the top 5 genes with the highest closeness centrality, while *LCP2* ranked among the top 5 in terms of degree score. These findings underscore the critical role of tyrosine protein kinases *HCK*, *LYN* and *SYK*, along with the signal transduction gene *STAT3*, in orchestrating microglia-mediated neuroinflammation in PD.

Furthermore, the topological analysis of the microglia PPI network involving the downregulated DEGs in PD, highlighted DEGs such as *CAMK2A*, *GRIN2B*, *GRM5* and *SYP*, which exhibited both high degree and closeness centrality. Moreover, *GRIN2A* ranked among the top 5 genes with the highest degree centrality, while *NRXN1* was among the top 5 in terms of closeness score. These findings emphasize the role of metabotropic (*GRM5*) and ionotropic glutamate receptors (*GRIN2B, GRIN2A*) in orchestrating microglia-mediated synaptic transmission in MDD, in contrast to PD where microglia exhibit a neuroinflammation phenotype, and the expression of these genes is downregulated.

### 3.2. Bulk comparative transcriptomic profiling of peripheral blood samples of PD and MDD

#### 3.2.1. Gene co-expression networks of PD and MDD

The gene co-expression networks of the GSE49126 (PD vs. Control), GSE72267 (PD vs. Control), and GSE39653 (MDD vs. Control) datasets, as shown in Supplementary File 3 Figures S1–S3, consisting of 375, 393, and 253 nodes, and 973, 886, and 360 edges, respectively. The top 200 upregulated and top 200 downregulated genes for each dataset can be found in Supplementary File 3 Tables S1–S3. Additionally, the cytoHubba (Chin et al., 2014) app was utilized for topological analysis, employing degree, closeness, betweenness, and MCC measures, to identify the highest-ranking genes within each co-expression network. The ranking tables and networks are available in Supplementary File 3 Tables S4–S15 and Supplementary File 3 Figures S4–S7. For the GSE49126 (PD vs. controls) dataset, the top-ranking genes identified are *COL5A1* (degree, closeness, and betweenness) and *MID1* (MCC). In the GSE72267 (PD vs. controls) dataset, *ZNF148* (degree, closeness, and betweenness) and *CD22* (MCC) are the top-ranking genes. Lastly, for the GSE39653 (MDD vs. controls) dataset, the top-ranking genes are *DENR* and *RNU1G2* (degree and MCC), and *CSTA* (closeness and betweenness).

In addition, the Venny tool (footnote 1) was employed to identify the shared, common, and distinct DEGs among the three datasets, as shown in Supplementary File 3 Table S16. *CD86* was identified as a common DEG among all three datasets. The *CD86* protein is expressed on antigen-presenting cells (APC), including dendritic cells, macrophages, Langerhans cells, B-cells, and memory B-cells (Lenschow et al., 1993). It provides co-stimulatory signals essential for T-cell activation and survival (Ohue and Nishikawa, 2019).

CytoHubba was also utilized to identify the top-ranking PPIs of *CD86*, based on degree, closeness, betweenness, and MCC topological measures. The results can be found in Supplementary File 3 Tables S17–S20 and Supplementary File 3 Figures S4–S7. The top-ranking proteins that *CD86* interacts with, based on the three topological measures, are *CD80* ➔ *CD4*➔ *CD86* ➔ *CTLA4* ➔ *CD8A* ➔ *IL10* ➔ *CD28* ➔ *CD247* ➔ *CD3E* ➔ *ICAM*. The order of proteins is based on the ranking obtained from cytoHubba, where nodes colored from red to yellow represent the highest to lowest ranked nodes based on the applied topological method.

#### 3.2.2. KEGG enrichment analysis and GO of PD and MDD

**Figure 5:**
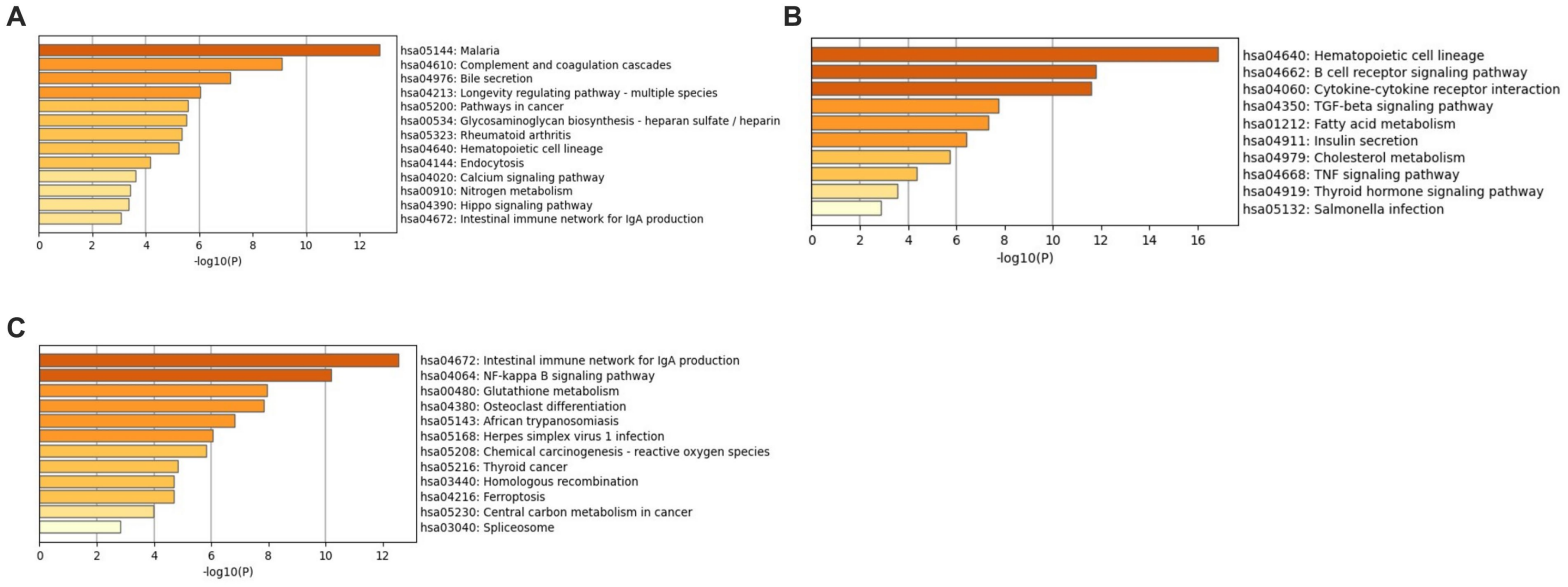
KEGG enrichment analysis with Metascape **(A)** GSE49126 (PD vs. controls) dataset pathways, **(B)** GSE72267 (PD vs. controls) dataset pathways and **(C)** GSE39653 (MDD vs. controls) dataset pathways.

Enrichment analysis was conducted using the KEGG and GO libraries with Metascape for each of the three datasets: GSE49126, GSE72267, and GSE39653. The top-ranked KEGG pathways for each dataset can be found in Supplementary File 4 Tables S1–S3. For the GSE49126 dataset (PD vs. Controls) (Figure 5A), the top three scoring pathways are as follows: (i) Malaria (hsa05144), (ii) Complement and coagulation cascades (hsa04610), and (iii) Bile secretion (hsa04976). In the GSE72267 dataset (PD vs. controls) (Figure 5B), the top three scoring pathways are: (i) Hematopoietic cell lineage (hsa04640), (ii) B cell receptor signalling pathway (hsa04662), and (iii) Cytokine-cytokine receptor interaction (hsa04060). Lastly, for the GSE39653 dataset (MDD vs. Controls) (Figure 5C), the top three scoring pathways are: (i) Intestinal immune network for IgA production (hsa04672), (ii) NF-kappa B signalling pathway (hsa04064), and (iii) Glutathione metabolism (hsa00480).

**Figure 6:**
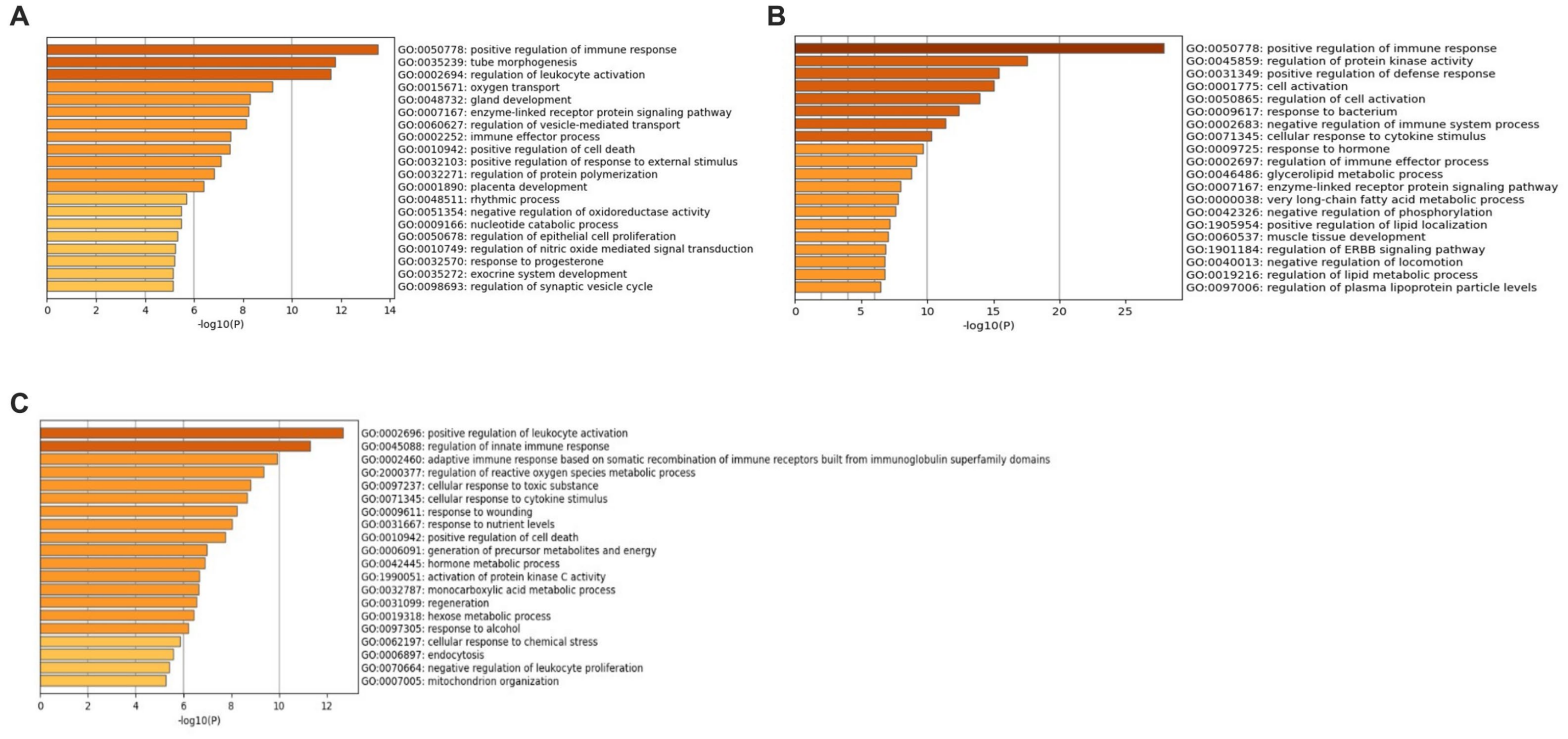
GO-BP terms with Metascape **(A)** GSE49126 (PD vs. controls) dataset GO-BP terms, **(B)** GSE72267 (PD vs. controls) dataset GO-BP terms and **(C)** GSE39653 (MDD vs. controls) GO-BP terms.

Additionally, GO analysis was performed using GO-BP, GO-CC, and GO-MF for all three datasets. The results of the GO-CC analysis can be found in Supplementary File 3 Figure S8 and Supplementary File 4 Tables S4–S6, and the GO-MF analysis in Supplementary File 3 Figure S9 and Supplementary File 4 Tables S7–S9. The GO-BP analysis (Figure 6A) for the GSE49126 dataset (PD vs. controls) revealed the top three GO terms as follows: (i) Positive regulation of immune response (GO:0050778), (ii) Tube morphogenesis (GO:0035239), and (iii) Regulation of leukocyte activation (GO:0002694). In the case of the GSE72267 dataset (PD vs. controls), the GO-BP analysis (Figure 6B) identified the top three GO terms as: (i) Positive regulation of immune response (GO:0050778), (ii) Regulation of protein kinase activity (GO:0045859), and (iii) Positive regulation of defence response (GO:0031349). For the GSE39653 dataset (MDD vs. controls), the top three GO terms from the GO-BP analysis (Figure 6C) were: (i) Positive regulation of leukocyte activation (GO:0002696), (ii) Regulation of innate immune response (GO:0045088), and (iii) Adaptive immune response based on somatic recombination of immune receptors built from immunoglobulin superfamily domains (GO:0002460).

The Venny tool (footnote 1) was utilized to identify the common and distinct KEGG pathways and GO terms among the three datasets: GSE49126, GSE72267, and GSE39653. The results of this analysis can be found in Supplementary File 3 Figure S10 and Supplementary File 3 Tables S1–S2.

## 4. Discussion

Proper functioning of the PFC is crucial for higher executive functions, such as decision-making, thinking, attention, emotion regulation, and impulse control. Dysfunctions in the PFC have been implicated in various neurological and NPDs, including PD and MDD (Xu et al., 2019). Interestingly, the presence of an NPD, such as MDD, can increase susceptibility risk of developing comorbid NDs like PD and Alzheimer’s disease (AD) (Wang et al., 2018; Jeong et al., 2021). Similarly, PD is associated with a higher incidence of comorbid MDD, which often results in more severe symptomatology (Marsh, 2013). However, the underlying pathophysiological mechanisms contributing to the comorbidity of PD and MDD remain poorly understood. Additionally, NPS are prevalent in NDs like AD, PD, and multiple sclerosis (MS) (Lyketsos et al., 2007). During the prodromal phase or early stages of NDs, patients may exhibit NPS that emerge before the onset of classical neurological symptoms. These emotional and behavioral symptoms during the early stages can lead to misdiagnosis, with approximately one-third of individuals being incorrectly diagnosed with NPDs rather than NDs, ultimately receiving inappropriate treatment (Woolley et al., 2011).

In this study, we aimed to compare the transcriptomic molecular signatures of PD and MDD. We accomplished this by integrating data obtained from both the analysis of snRNA and bulk mRNA data in the brain and peripheral tissues, respectively. The integration of these two omics levels offers a more comprehensive understanding of the shared and distinct molecular pathophysiology of PD and MDD, enabling exploration of these conditions not only in the periphery but also at the brain level. This provides an enhanced resolution and an accurate view of their molecular signatures, potentially leading to the identification of more effective diagnostic markers.

Comparative analysis of the snRNA data from post-mortem dlPFC (BA9) tissues enabled us to identify specific differences in cell types and their gene expression patterns between PD and MDD. Our approach aimed at providing meaningful biological insights into both disorders, utilizing a condition-specific methodology that focused on differentiating distinct and shared clusters and cell types between MDD and PD, rather than conducting separate comparisons with control groups. In this single-cell analysis, we directly compared PD with MDD, rather than conducting separate analyses for PD versus control and MDD versus control, followed by a comparison of the two conditions. This choice aimed to minimize potential biases or inaccuracies stemming from comparing datasets with different experimental conditions, platforms, or methodologies. This approach offers several advantages. By directly comparing PD and MDD, both conditions underwent the same data processing steps, reducing the influence of potential confounding factors. Additionally, directly comparing PD and MDD allowed us to concentrate on disease-specific features, aiding in the identification of unique cellular and molecular characteristics associated with each condition. This enabled us to shed light on the distinct pathophysiological mechanisms underlying PD in comparison to MDD in the dlPFC. Finally, a direct comparison of PD and MDD can be more biologically relevant, particularly when the research objective, as in our study, was to elucidate the potential distinct or shared clusters, cell types and mechanisms that characterize the two conditions. This might not have been apparent when comparing each condition individually to control groups.

Since we conducted a direct comparison between MDD and PD, which differs from the approach taken in the original studies where control groups were included, the annotation of our clusters incorporates components from the cluster annotations of both original papers. For instance, in line with the original paper of the MDD dataset, we identified 26 clusters, maintaining consistency with the previous annotations. This approach ensures that we align with the existing annotations while emphasizing the direct comparison of MDD and PD. Furthermore, in alignment with the original paper of the PD dataset, we identified the presence of T cells. However, it’s worth noting that this particular cell type was not identified in our annotation of the MDD dataset, which is also consistent with the original paper of the MDD dataset. Thus, the annotation of our clusters correctly incorporates aspects from both original studies to facilitate a meaningful comparison between MDD and PD.

Our approach, which involved directly comparing PD and MDD cells at the single-cell level, revealed the presence of seven common cell types in the dlPFC. However, it’s important to note that their relative proportions differed between the two conditions. PD exhibited a significantly higher proportion of immune system cells, with MDD showing very few microglia cells and a complete absence of T cells, indicating the presence of neuroinflammation in the dlPFC of PD patients but not MDD. Additionally, compared to MDD, PD showed a higher relative proportion of astrocytes, OPCs, and oligodendrocytes, suggesting the presence of astrogliosis, demyelination, and remyelination, respectively, in PD but not MDD. Previous studies have demonstrated higher myelin water fraction in frontal brain regions of PD patients, indicating alterations in myelin content (Dean et al., 2016; Isaacs et al., 2019). Furthermore, MDD exhibited a significantly higher proportion of excitatory neurons (23,732 nuclei) compared to inhibitory neurons (7,823 nuclei), as well as a higher proportion of excitatory neurons compared to PD (10,346 nuclei). In the healthy mammalian cortex, the overall ratio of excitatory to inhibitory (E/I) neurons is approximately 80:20, although it can vary depending on the region and age (Dean et al., 2016; Isaacs et al., 2019). Based on our results, the E/I ratio in MDD is approximately 75:25, which is relatively close to the normal ratio, suggesting only a slight increase in inhibitory inputs in MDD. However, in PD, the E/I ratio is 62:38, indicating increased inhibition in the dlPFC of PD patients. Our findings suggest that neuroinflammation, increased inhibition, astrogliosis, and alterations in myelin content in the dlPFC contribute to the observed non-motor symptoms of PD, including cognitive dysfunction and NPS (Rubenstein and Merzenich, 2003).

Additionally, we conducted differential expression analysis to profile the molecular signatures of each of the seven common cell types between the two conditions. We identified 466 upregulated and 954 downregulated DEGs in PD compared to MDD. Notably, microglia exhibited the most significant difference in gene expression profiles, accounting for almost half (42%) of the observed differences in all cells. To gain insights into the biological functions disrupted in each cell type, we performed GO enrichment analysis on the upregulated and downregulated DEGs identified when comparing PD to MDD. The analysis revealed that microglia in PD displayed a pro-inflammatory phenotype, characterized by the upregulation of Toll-like receptor 2 (TLR2) and Toll-like receptor 5 (TLR5) gene expression. Increasing evidence supports the role of Toll-like receptors (TLRs) in inducing neuroinflammation in various NDs, including PD, particularly TLR2 (Fiebich et al., 2018; Heidari et al., 2022). It has been shown that α-synuclein aggregates activate the Nod-like receptor family pyrin domain containing 3 (NLRP3) inflammasome through interactions with TLR2 and TLR5, leading to the release of pro-inflammatory cytokines and increased neuroinflammation, thus contributing to disease progression (Li et al., 2021; Scheiblich et al., 2021). Contrary to PD, microglia in MDD appeared to be involved in the regulation of chemical synaptic transmission and trans-synaptic signaling, potentially through oligodendrocyte-microglia crosstalk. These processes were also found to be downregulated in oligodendrocytes and OPCs in PD but upregulated in MDD. Oligodendrocyte-microglia crosstalk is essential for maintaining brain homeostasis, including synapse formation and transmission, remyelination, and immune regulation (Peferoen et al., 2014; Kalafatakis and Karagogeos, 2021). Oligodendrocytes not only contribute to myelin formation but also regulate immune responses through the production of immune-regulating factors and act as antigen-presenting cells (APCs) for T cell activation (Peferoen et al., 2014; Kalafatakis and Karagogeos, 2021). Thus, based on the single-cell dlPFC data, PD and MDD exhibit distinct patterns of microglia-oligodendrocyte/OPCs communication.

Based on the DEGs and biological functions results, there are notable differences in the function of microglia cells between PD and MDD conditions. To further investigate these differences, we constructed and analyzed microglia cell-specific PPI networks using the DEGs found to be upregulated and downregulated in the comparison between PD and MDD microglia cells. In the microglia cell-specific PPI network constructed using the upregulated DEGs, topological analysis revealed several key genes with high centrality that exhibited upregulation in PD microglia. These genes included *STAT3*, *LYN*, *SYK*, *GRB2*, *HCK*, and *LCP2*, all of which play crucial roles in orchestrating signaling pathways associated with inflammation and immune responses. Importantly, *in vitro* and *in vivo* evidence has demonstrated that exposure to α-synuclein activates the JAK/STAT pathway in microglia, leading to their activation (Qin et al., 2016). Furthermore, pharmacological inhibition of this pathway has been shown promise in protecting against α-synuclein-induced neuroinflammation and the subsequent neurodegeneration of dopaminergic neurons (Qin et al., 2016).

Conversely, topological analysis of the microglia cell-specific PPI network, which was constructed using the downregulated DEGs identified when comparing PD to MDD, revealed high centrality genes such as *CAMK2A*, *GRIN2B*, *GRM5*, *SYP*, *GRIN2A*, and *NRXN*. These genes are closely associated with synaptic transmission, specifically glutamate receptor signaling. Microglia cells are known to express several receptors for neurotransmitters, including for glutamate (Liu et al., 2016). Activation of these receptors on microglia can modulate the release of neuroactive molecules, such as free radicals, chemokines and cytokines, which can have either neuroprotective or neurotoxic effects (Liu et al., 2016). For instance, the activation of metabotropic glutamate receptor 5α, encoded by the *GRM5* gene, has been shown to reduce microglial tumor necrosis factor α (TNFα) production, thereby inhibiting microglia-mediated neuroinflammation and neurotoxicity (Byrnes et al., 2009). Additionally, evidence suggests that GluN2A receptor on microglia, encoded by *GRIN2A* gene, plays a role in regulating microglia–neuron physical interactions (Eyo et al., 2018). Through this interaction, microglia can influence neuronal activity, synapse formation, survival, and remodeling (Badimon et al., 2020).

These findings underscore significant differences in the high centrality DEGs of microglia between PD and MDD. In PD microglia, we observe high centrality nodes that promote a pro-inflammatory and immune-activating state. Conversely, in MDD microglia, the high centrality genes are associated to synaptic transmission. This divergence suggests that PD is characterized by microglia-mediated alterations in synaptic plasticity and neurotransmission, coupled with a neuroinflammatory phenotype, which together may contribute to the observed synaptic dysfunction and neurotoxicity in PD. These differences provide valuable insights into the distinct pathophysiological mechanisms underlying microglia in PD versus MDD.

Furthermore, analysis of bulk mRNA data from peripheral blood of PD and MDD patients identified *CD86* as a shared DEG between the two conditions. Our analysis also revealed the top-ranking PPIs of *CD86* based on three topological measures to be: *CD80* ➔ *CD4*➔ *CD86* ➔ *CTLA4* ➔ *CD8A* ➔ *IL10* ➔ *CD28* ➔ *CD247* ➔ *CD3E* ➔ *ICAM1,* arranged according to the cytoHubba ranking order. Interestingly, *CD86* and *CD80* act as ligands for *CD28* on the surface of naive T cells and the inhibitory receptor *CTLA-4* (Linsley et al., 1991). *CD28* and *CTLA-4* play vital but opposing roles in T cell stimulation, with *CD28* promoting T cell response and *CTLA-4* inhibiting it (Linsley et al., 1991). *CD4* is found on T helper cells (Th1, Th2, Th17), *CD4^+^* regulatory T cells, and binds to the major histocompatibility complex (MHC) class II molecules expressed on APCs such as B cells, macrophages, and dendritic cells (Safran et al., 2010). *CD8* glycoprotein, composed of *CD8α* and *CD8β* subunits, is expressed on *CD8^+^* cytotoxic T lymphocytes and *CD8^+^* regulatory T cells, which are crucial for host immune responses against viral infections and cancer cells. *CD8* binds to MHC class I molecules displayed by APCs to enhance T cell signaling. *CTLA4* is located on T cells and is responsible for maintaining immune homeostasis. *CD3E* is part of the TCR-CD3 complex, which plays an important role in antigen recognition and several signal transduction pathways. The anti-inflammatory molecule *IL-10* was also identified in the PPI network. *CD247* plays a role in coupling antigen recognition to numerous intracellular signal transduction pathways. Additionally, low antigen expression results in an impaired immune response. Finally, *ICAM1 (CD54)* mediates adhesion of T cells with APCs and is also involved in T cell-to-T cell and T cell-to-B cell interactions (Safran et al., 2010).

The cell markers, cytokines, and interleukins identified in our analysis as being involved in PD and MDD pathophysiology have also been investigated in previous studies. Specifically, studies (Hisanaga et al., 2001) have shown that the continuous increase of these immune cells may indicate post-infectious immune abnormalities that are likely associated with PD pathogenesis or psychological distress (depression, stress, or anxiety), contributing to disease progression and severity in both NDs and NPDs. Moreover, dysfunction of regulatory T lymphocytes and a shift towards a pro-inflammatory phenotype (Thome et al., 2021), can result in the loss of Treg suppression, which may enhance PD progression. Conversely, enhancing Treg suppressive function may be a potential therapeutic avenue for PD patients as well as for other NDs and NPDs. In addition, a study by Chen et al. (2021) investigated the percentage of T-cell subsets and immunoglobulins in the serum of PD patients. The study indicated that PD patients have increased levels of CD3^+^ and CD4^+^ T-cells compared to healthy controls, as well as a significantly higher CD4/CD8 ratio. However, PD patients and controls showed similar CD8^+^ T-cell percentages. Furthermore, CD4^+^ T-cells were found to be inversely correlated with the Hoehn and Yahr Staging Scale, which is used to describe symptom progression in PD, while IgG was positively correlated with disease duration and the Unified Parkinson’s Disease Rating Scale (UPDRS) section III. These findings suggest escalated immune activity in the periphery in PD, and alterations in CD4^+^/CD8^+^ and IgG levels indicate an active role of peripheral immunity in PD progression (Chen et al., 2021).

Interestingly, alterations in cell immunity have also been associated with MDD pathophysiology. Evidence suggests that immunological dysfunction in MDD may not necessarily be involved in its development but may be associated with specific features of the disease (Euteneuer et al., 2017). The same study examined the association between different subtypes of depression and characteristics such as melancholic vs. non-melancholic depression, chronic vs. non-chronic depression, age of onset, and cognitive and somatic symptoms. They investigated C-reactive protein (CRP), interleukin 6 (IL-6), interleukin 10 (IL-10), and various leukocyte populations (lymphocytes, neutrophils, monocytes, T-helper and cytotoxic T-cells, B cells, and natural killer (NK) cells; Euteneuer et al., 2017) by obtaining plasma from MDD patients and controls. Based on their results, MDD patients demonstrated increased CRP concentration, neutrophils and monocytes, and neutrophil/lymphocyte (NLR) ratio (Euteneuer et al., 2017). MDD patients also showed lower IL-10 concentrations, which were associated with severe somatic symptoms.

The KEGG enrichment analysis has identified various immune pathways for both the PD and MDD datasets. Specifically, some of the pathways identified include the complement & coagulation cascade (hsa 04060), B-cell receptor signaling (hsa 04662), cytokine-cytokine interaction (hsa 04060), intestinal immune network for IgA production (hsa 04672), and NF-kappa B signaling (hsa 04064). These findings suggest an important role of peripheral immune system activation in both PD and MDD. The enteric system which is the largest lymphoid tissue in the body, plays a prominent role in enteric immunity. This system has the ability to generate a large concentration of anti-inflammatory immunoglobulin A (IgA) (hsa 04672) antibodies, serving as the first line of defense against invading pathogens (Zeng et al., 2022).

Emerging evidence suggests the importance of gastrointestinal tract (GIT) dysfunction and supports the hypothesis that PD pathogenesis may originate from the gut (Warnecke et al., 2022). Numerous PD patients suffer with GIT-related symptoms, such as dysphagia, vomiting, constipation, nausea and bloating during disease progression (Warnecke et al., 2022). Lewy bodies, the histological hallmark of PD, can be transported to the CNS via the vagus nerve. Additionally, the altered composition of gut microbiota results in an imbalance between beneficial and harmful microbial metabolites, which in turn interacts with increased gut permeability and inflammation (Zeng et al., 2022). This activated inflammatory response subsequently affects the CNS resulting in PD pathology (Zeng et al., 2022).

A previous study conducted by Brown et al. (2023), investigated the IgA biome profiles and their correlation with clinical PD subtypes. The study utilized stool samples from akinetic rigid (AR) or tremor dominant (TD) PD patient subtypes to identify unique taxa associated with each clinical phenotype (Brown et al., 2023). IgA Biome analysis revealed significant differences in both alpha and beta diversity between PD phenotypes (Brown et al., 2023). Furthermore, the study found that the *Firmicutes*/*Bacteroides* ratio was higher in the TD phenotype in comparison to the AR phenotype. Taxa analysis further identified a pro-inflammatory bacterial profile in the IgA^+^ fraction of AR PD subtype compared to the TD subtype (Brown et al., 2023). Interestingly, a study conducted by our group, using network-based bioinformatics approaches, demonstrated that microbiota, through their metabolic products, have the capacity to affect humoral immune response mediated by circulating immunoglobulins (Onisiforou and Spyrou, 2022). This finding aligns with existing evidence demonstrating that commensal gut microbiota can influence antibody production, particularly IgA (Kim and Kim, 2017). One can argue that special attention is needed to research and comprehend the connections between PD, the microbiota, dysfunction of the enteric system, and the anti-inflammatory role of IgA in PD.

Emerging evidence supports the concept of bi-directional communication between enteric microbiota, endocrine and immune systems, in mediating key nervous system process such as neuroinflammation, neurotransmission, neurogenesis and activation of the stress axes (Cruz-Pereira et al., 2020). In the field of research, there is growing appreciation of the significance of the microbiota-gut-brain axis and its role in the pathology of depression (Cruz-Pereira et al., 2020).

There are numerous studies (Cruz-Pereira et al., 2020) indicating alternations in the composition of microbiota in individuals with depression individuals. These studies have observed that the abundance of *Faecalibacterium* is negatively correlated with symptom severity (Cruz-Pereira et al., 2020). One observational study revealed that challenging the enteric microbiota with either single or recurrent antibiotic treatment elevated the risk of anxiety and depression (Cruz-Pereira et al., 2020). Most recently, a large population study demonstrated that *Coprococcus* and *Dialister* microbiota strains were predictors of a healthier quality of life but were frequently depleted in untreated depressive patients, while *Butyricicoccu* microbiota was correlated with anti-depressant treatment (Cruz-Pereira et al., 2020). Further research is required to understand the mechanisms underlying the interactions between microbiota and depression, as well as how dietary changes can affect the microbiota environment in treated and untreated MDD patients.

There is growing interest in the prospective role of the immune system in the pathogenesis of PD. Genome-wide association studies (GWASs) have linked haplotypes of the major histocompatibility complex (MHC) class II genes and numerous other immune-related gene (*TLR9*, *IL-1R2, SATB1, STAB1, GBA, CD38, CD19, NOD2*, and *FYN*) to an increased risk of developing the disease (Li et al., 2022). CD4^+^ T cell infiltration and deposition of IgG have been observed in post-mortem brain tissues of PD mouse models and patients, implicating both humoral and cellular immune responses (Li et al., 2022). However, there are limited studies investigating the role of B-cells and the B-cell receptor signaling pathway (hsa 04662) in PD pathogenesis.

A previous study, by Scott et al. (2023) investigated B-cell response in PD patients. They measured alpha-synuclein and tau antibodies in the serum of PD patients with rapid eye movement sleep behavior disorder (REM-SBD), early PD, and matched controls. Furthermore, they measured B-cell activating factor of the tumor necrosis factor receptor family, C-reactive protein and total IgA. The study observed increased levels of antibodies against a-synuclein fibrils in REM-SBD patients (Scott et al., 2023). This suggests that an early humoral response to alpha-synuclein occurs prior to the development of PD. Furthermore, B lymphocyte phenotyping was performed in early PD patients and their matched controls. They found that PD patients at a higher risk of developing early dementia had a decrease in their B-cells (Scott et al., 2023). On the other hand, PD patients who had a higher proportion of regulatory B-cells had greater motor scores, suggesting a possible protective role of these cells in PD.

In contrast, B cells isolated from PD patients at a greater risk of dementia exhibited increased levels of IL-6 and IL-10. Additionally, peripheral blood lymphocytes in an alpha-synuclein transgenic PD mouse model showed a decrease in B-cells, suggesting a potential association with alpha-synuclein pathology (Scott et al., 2023). In a toxin-based PD mouse model, B-cell depletion was associated to worsening of behavioral and pathological outcomes, supporting the assumption that B-cells play an early protective role in dopaminergic (DA) cell loss (Scott et al., 2023). Therefore, regulatory B-cells could have a protective role in PD mouse models, possibly by attenuating inflammation and DA cell loss. As a results, further investigation of B-cells is warranted, as they could be considered as a potential therapeutic target (Scott et al., 2023). It is well-established that dysregulation of both innate and adaptive immune responses occurs in MDD patients, which can hinder prognosis, including the response to anti-depressant treatments (Beurel et al., 2020). Altered B-cell homeostasis has been observed in MDD patients, and these patients often exhibit cell-mediated immune responses and pro-inflammatory activity (Scott et al., 2023). Besides their well-known role as antibody producers, B-cells play a critical role in inflammatory responses by secreting both pro- and anti-inflammatory factors (Scott et al., 2023). A study by Ahmetspahic et al. (2018) characterized B-cells at distinct developmental stages such as (i) transitional, (ii) naïve-mature, (iii) antigen experienced, (iv) non-switched memory cells, (v) plasmablasts and (vi) regulatory B-cells. They conducted a six-week follow-up of circulating B-cells in MDD patients responding to therapy and non-responders. The study found that the prevalence of naïve lgD^+^CD27^−^ B cells, but not lgD^+^CD27^+^ memory B cells, were lower in severely depressive patients in comparison to healthy controls or mildly/moderately depressed patients (Ahmetspahic et al., 2018). Moreover, B-cells with immune-regulatory roles including CD1d^+^CD5^+^ B cells and CD24^+^CD38^hi^ and transitional B-cells were shown to be decreased in depressive patients (Ahmetspahic et al., 2018). Furthermore, a reduction of CD5 surface expression on transitional B-cells was correlated with severe depression. These fundings suggest that immune cells, including B-cells, may be potential biomarkers for subtyping depression and patient stratification in future clinical trials for depression.

To the best of our knowledge, our study represents the first integration of bulk and single-cell transcriptomics data to investigate CNS diseases using two distinct tissues; dlPFC and blood. While limitations exist due to the small number of single-cell datasets available, our work provides valuable insights for identifying potential pharmacotherapies targeting the distinct tissue pathophysiological abnormalities observed in MDD and PD. Future research may explore multi-target drugs and pharmacotherapeutic strategies aimed at inflammatory cytokine signaling, immune pathways, and the impact of cytokines on neurotransmitters. These innovative approaches could offer novel ways to prevent, delay, or even reverse the effects of cytokines and immune cells on individuals suffering from MDD and PD.

## Data availability statement

The original contributions presented in the study are included in the article/Supplementary material, further inquiries can be directed to the corresponding author.

## Author contributions

CCC: Conceptualization, Data curation, Formal analysis, Investigation, Methodology, Resources, Software, Visualization, Writing – original draft, Writing – review & editing. AO: Conceptualization, Data curation, Formal analysis, Funding acquisition, Investigation, Methodology, Resources, Software, Validation, Visualization, Writing – original draft, Writing – review & editing. PZ: Supervision, Writing – review & editing. EZP: Supervision, Writing – review & editing.

## Footnotes

^1^ https://bioinfogp.cnb.csic.es/tools/venny/
