# Supplementary material for: Unraveling the transcriptomic signatures of Parkinson’s disease and major depression using single-cell and bulk data
Source: Front Aging Neurosci. 2023 Nov 7;15:1273855. doi: 10.3389/fnagi.2023.1273855 (PMC10664927; doi:10.3389/fnagi.2023.1273855)
Supplement: Supplementary file 1 [file Supplementary_Material.zip › Supplementary_Material/Supplementary File 1.docx]

Unraveling the transcriptomic signatures of Parkinson’s Disease and Major Depression using single-cell and bulk data

Christiana C. Christodoulou, Anna Onisiforou, Panos Zanos, Eleni Zamba Papanicolaou

**Supplementary File 1 : Analysis of single cell data from MDD and PD**

**
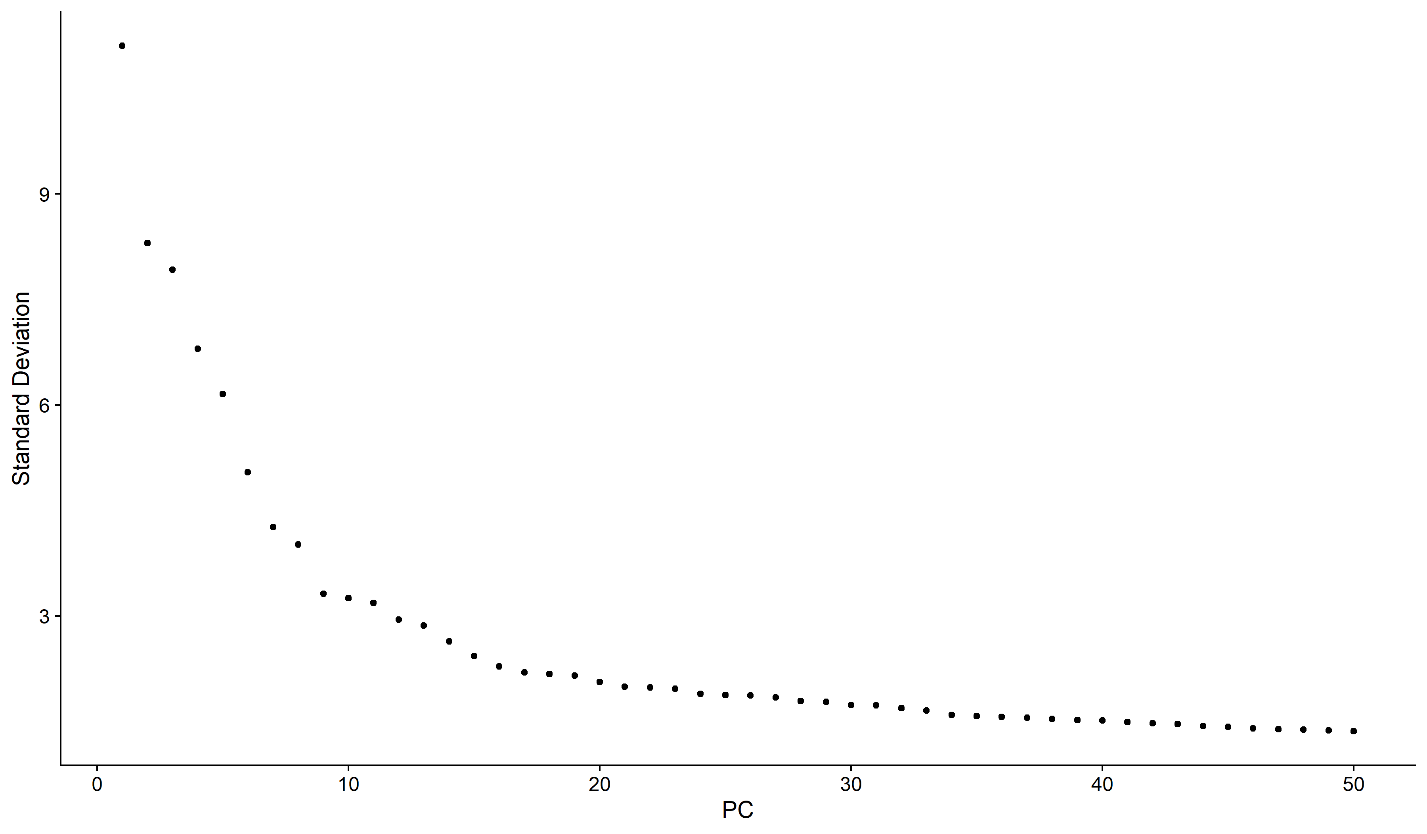
**

**Supplementary Figure 1:** Elbow plot visualization of the standard deviation of the first 50 PC of the integrated dataset of MDD and PD.

**
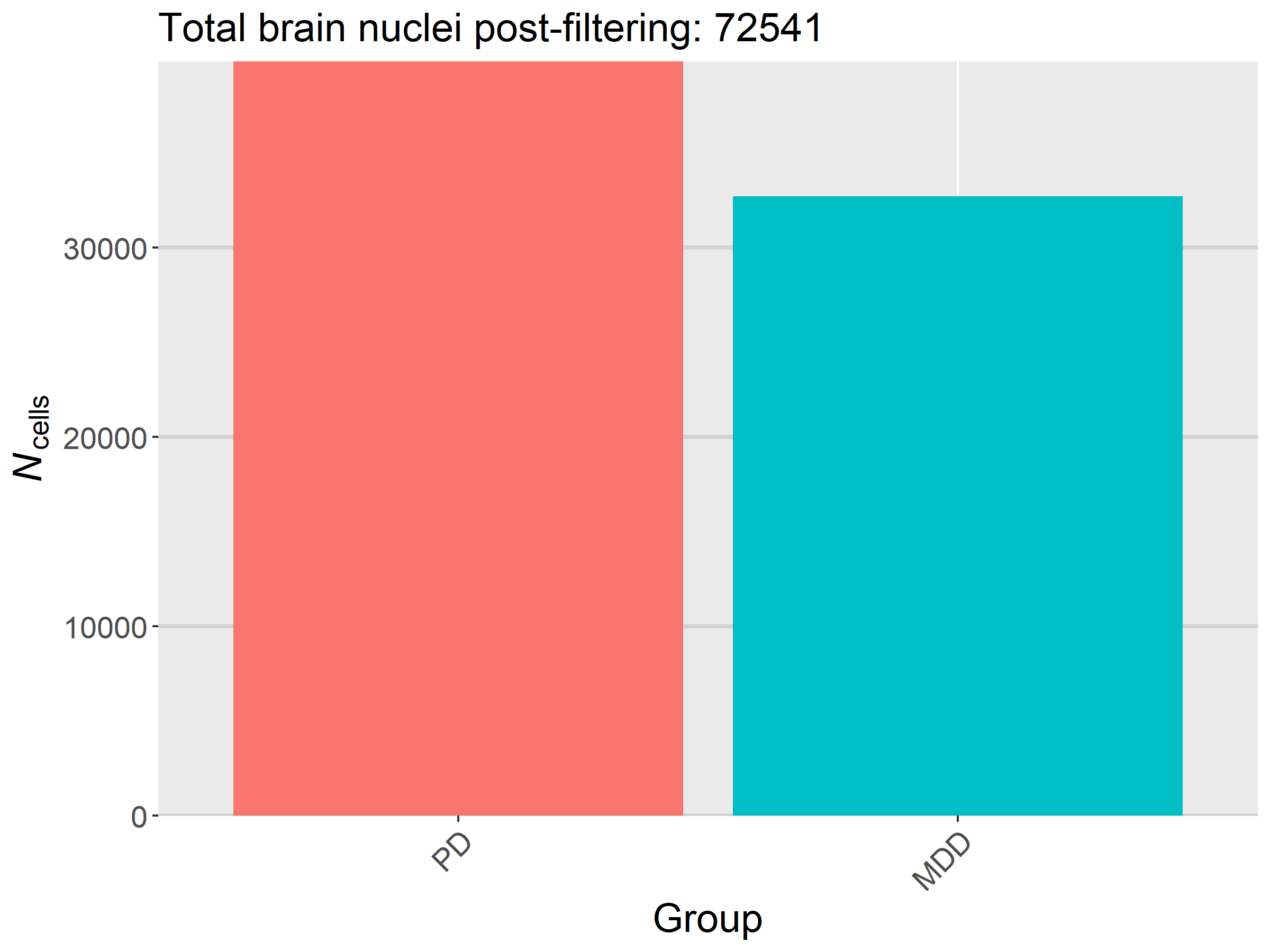
**

**Supplementary Figure 2:** Plot indicating the total number of brain nuclei post-filtering from PD and MDD that were used for clustering.


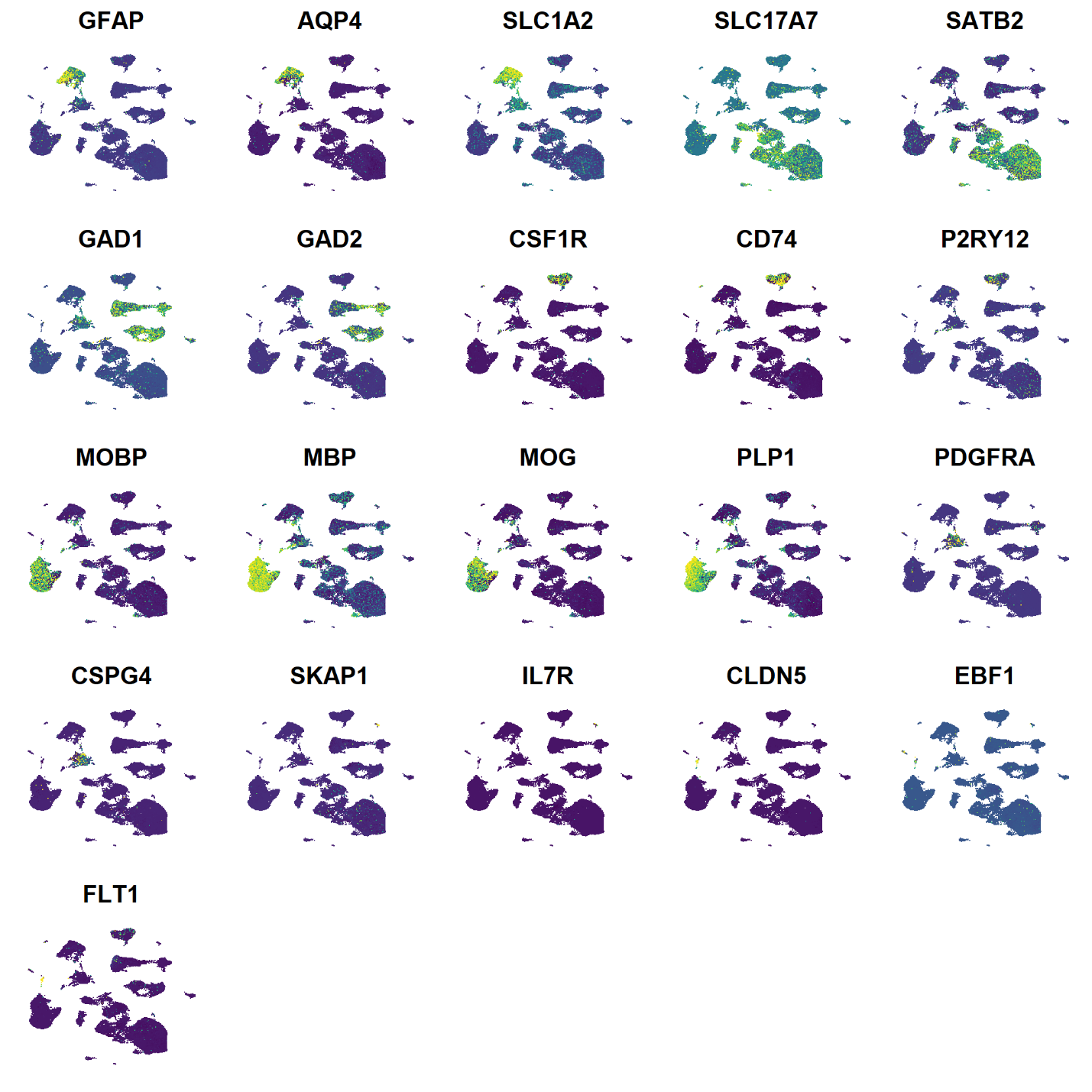


**Supplementary Figure 3:** Basic canonical cell marker feature plot of each of the cell markers used to identify the eight cell types and annotate the 26 clusters of the integrated dataset of PD and MDD.

**Supplementary Table 1:** Number of nuclei from PD and MDD found in each annotated cluster.

|  | **MDD** | **PD** |
| --- | --- | --- |
| **ASTRO-20** | 87 | 261 |
| **ASTRO-4** | 534 | 3841 |
| **ENDO-21** | 3 | 266 |
| **ENDO-23** | 32 | 191 |
| **EX-0** | 9382 | 6068 |
| **EX-12** | 2238 | 280 |
| **EX-14** | 1545 | 165 |
| **EX-15** | 1158 | 265 |
| **EX-17** | 8 | 697 |
| **EX-18** | 370 | 130 |
| **EX-24** | 46 | 140 |
| **EX-5** | 3620 | 690 |
| **EX-6** | 3490 | 617 |
| **EX-9** | 1875 | 1294 |
| **INH-11** | 1302 | 1301 |
| **INH-13** | 1099 | 711 |
| **INH-16** | 362 | 357 |
| **INH-19** | 150 | 262 |
| **INH-2** | 2637 | 2649 |
| **INH-8** | 2273 | 949 |
| **MIGRO-25** | 0 | 132 |
| **MIGRO-7** | 60 | 3639 |
| **OLIGO-1** | 90 | 6621 |
| **OLIGO-3** | 22 | 5255 |
| **OPC-10** | 324 | 2799 |
| **TCELL-22** | 0 | 254 |
