# Supplementary material for: Unraveling the transcriptomic signatures of Parkinson’s disease and major depression using single-cell and bulk data
Source: Front Aging Neurosci. 2023 Nov 7;15:1273855. doi: 10.3389/fnagi.2023.1273855 (PMC10664927; doi:10.3389/fnagi.2023.1273855)
Supplement: Supplementary file 1 [file Supplementary_Material.zip › Supplementary_Material/Supplementry File 3.docx]

**Comparison of the molecular signature of Parkinson’s Disease and Major Depressive Disorder using single-cell and bulk transcriptomic data**

**Christiana C. Christodoulou, Anna Onisiforou, Panos Zanos, Eleni Zamba Papanicolaou**

**Supplementary File 3**

**
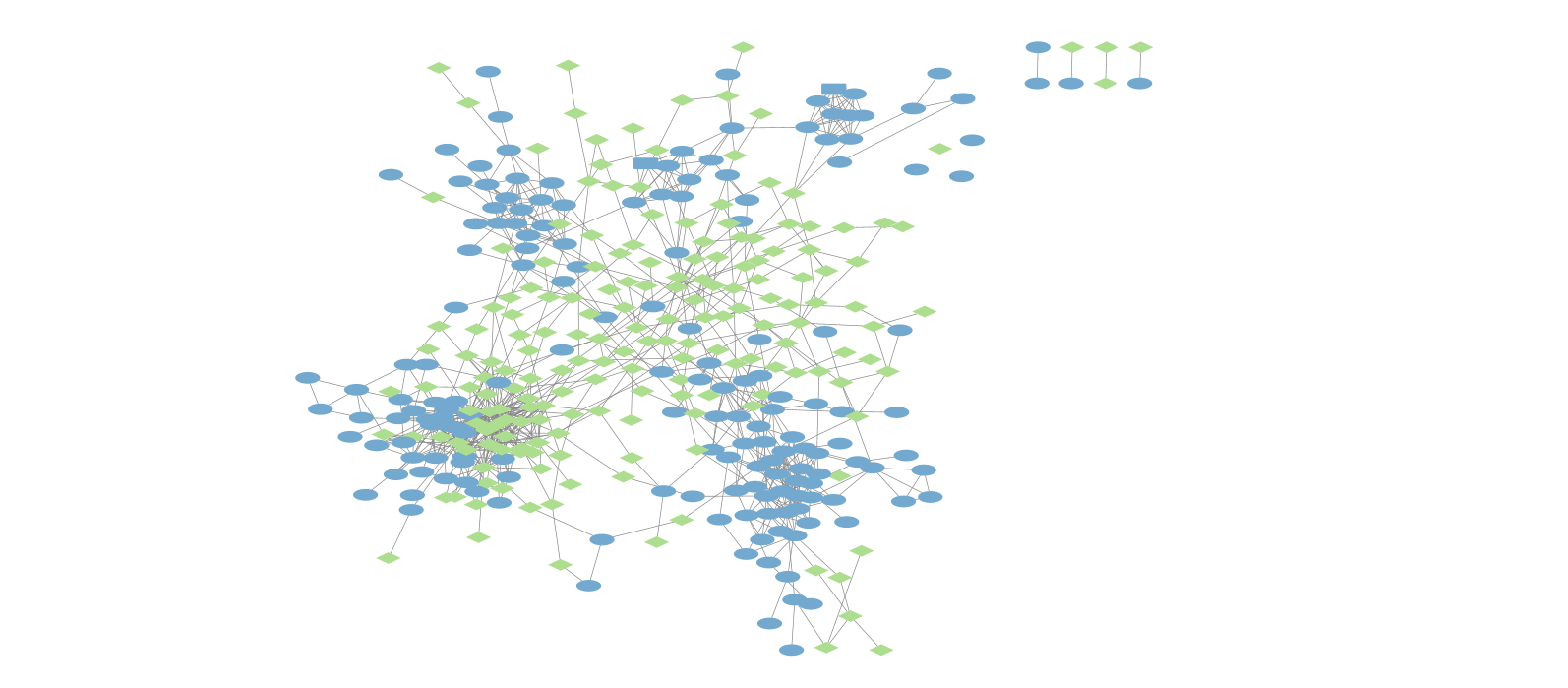
**

**Supplementary Figure 1:** Gene co-expression network of GSE49126.


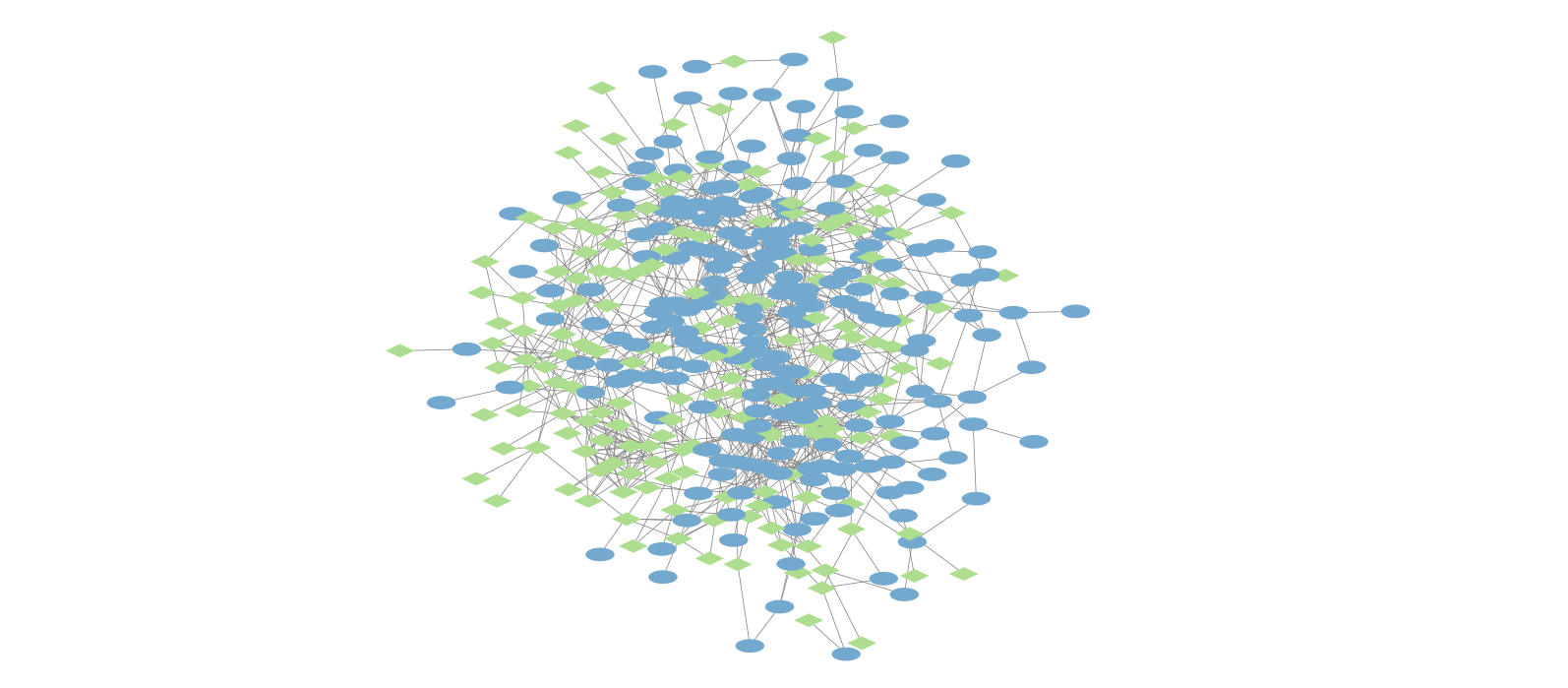


**Supplementary Figure 2:** Gene co-expression network of GSE72267.


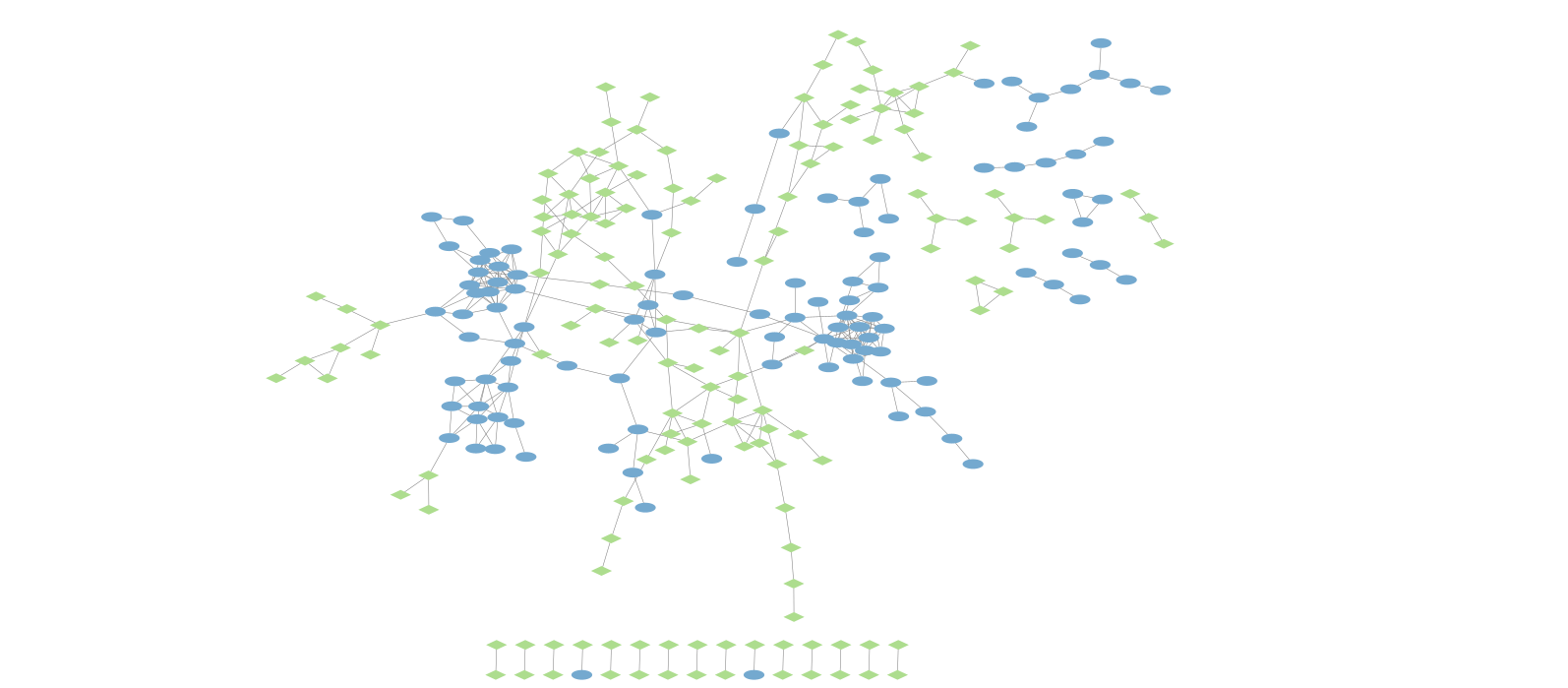


**Supplementary Figure 3:** Gene co-expression network of GSE39653.


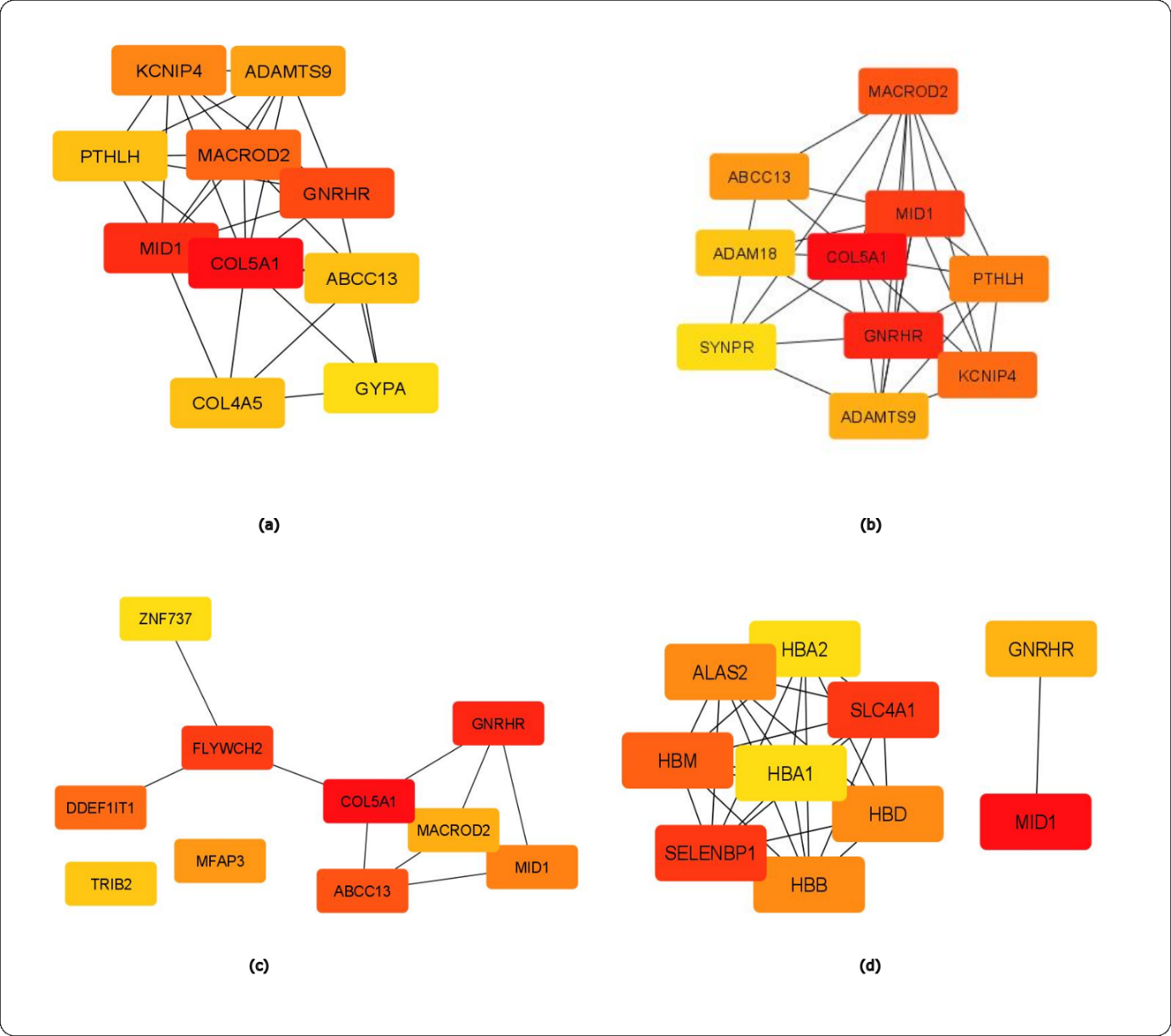


**Supplementary Figure 4: Top-ranking genes of GSE49126 co-expression network using cytoHubba. (A)** Degree, **(B)** Closeness**, (C)** Betweenness and **(D)** MCC.


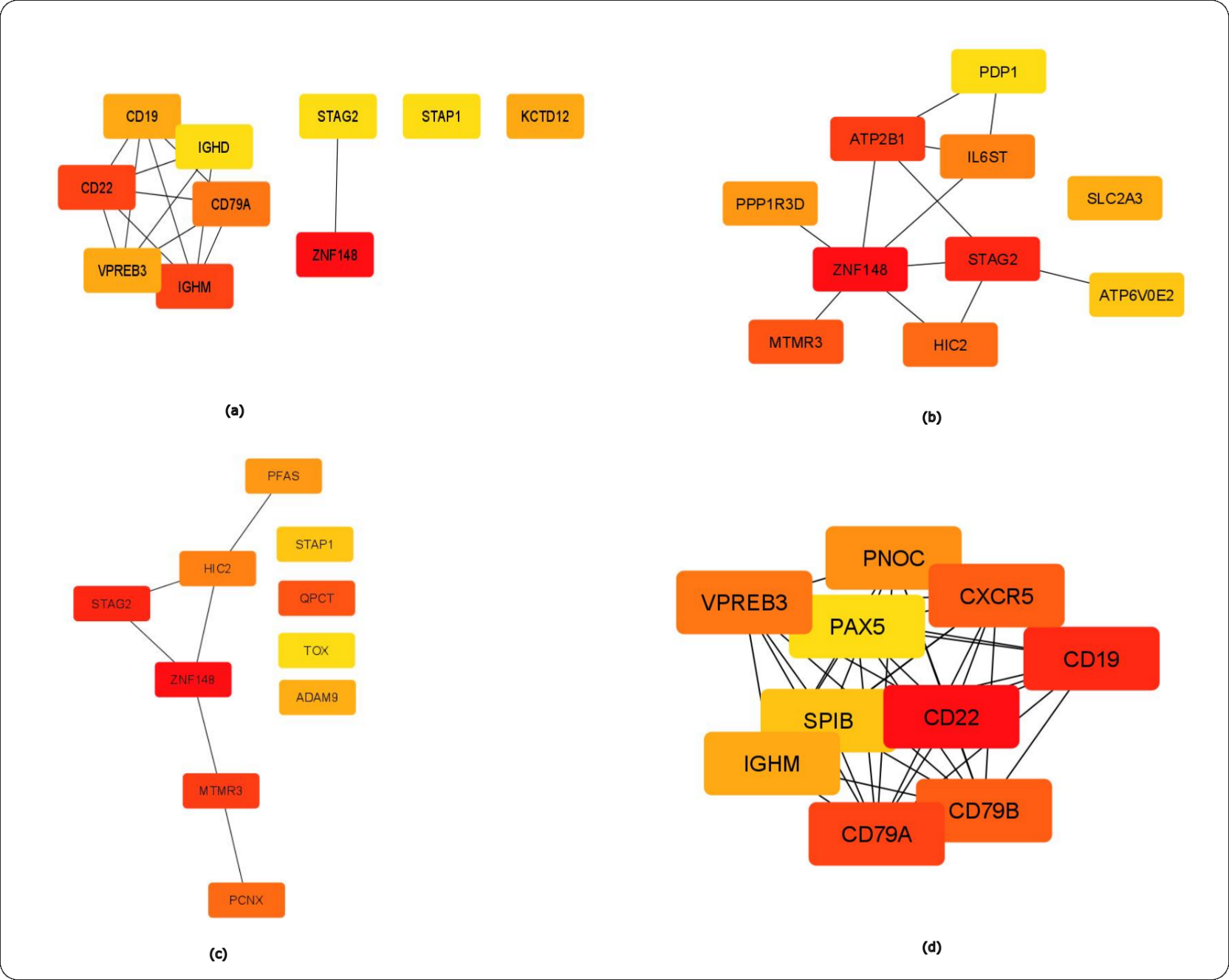


**Supplementary Figure 5: Top-ranking genes of GSE72267 co-expression network using cytoHubba. (A)** Degree, **(B)** Closeness**, (C)** Betweenness and **(D)** MCC.


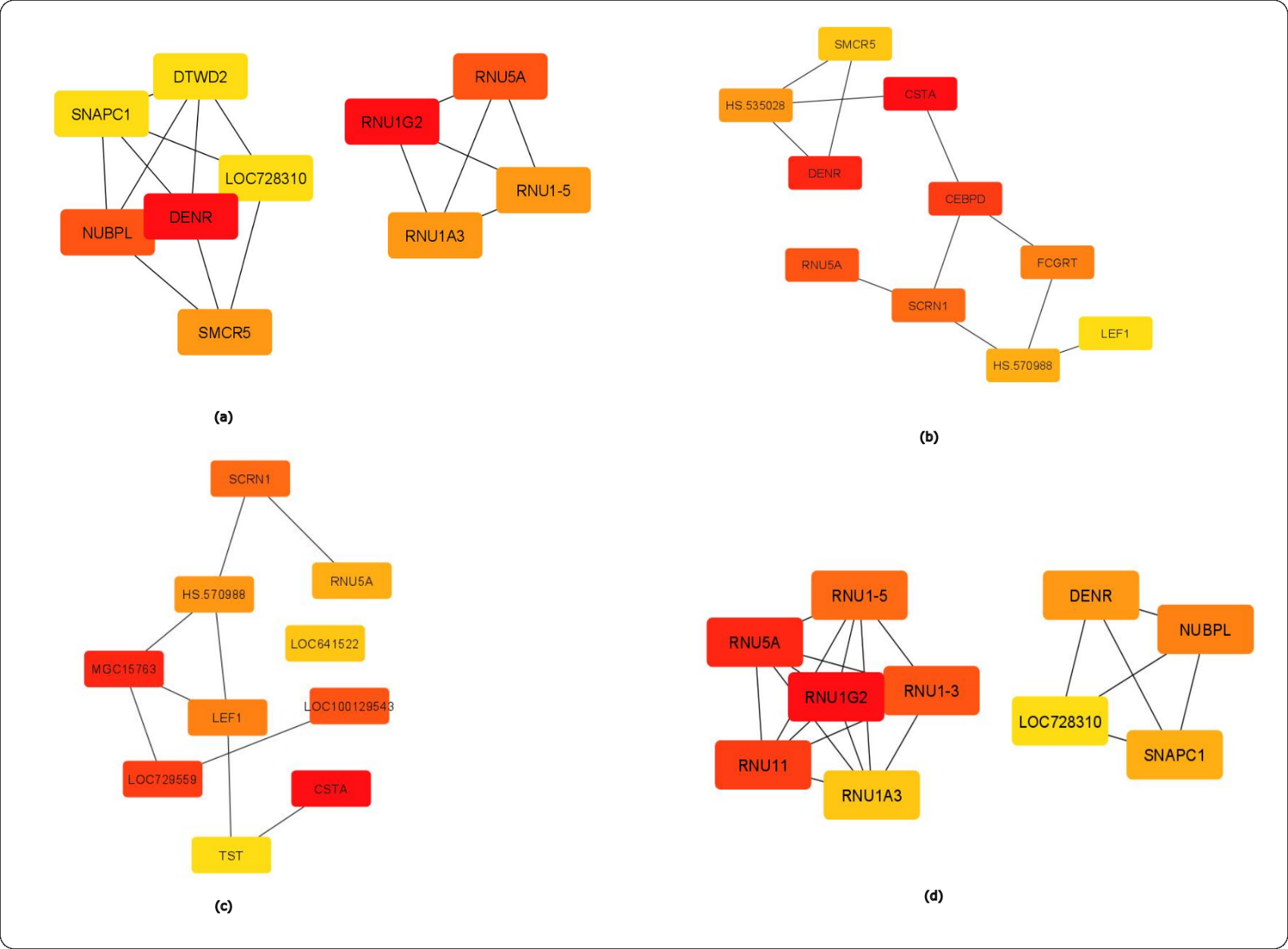


**Supplementary Figure 6: Top-ranking genes of GSE39653 co-expression network using cytoHubba. (A)** Degree, **(B)** Closeness**, (C)** Betweenness and **(D)** MCC.


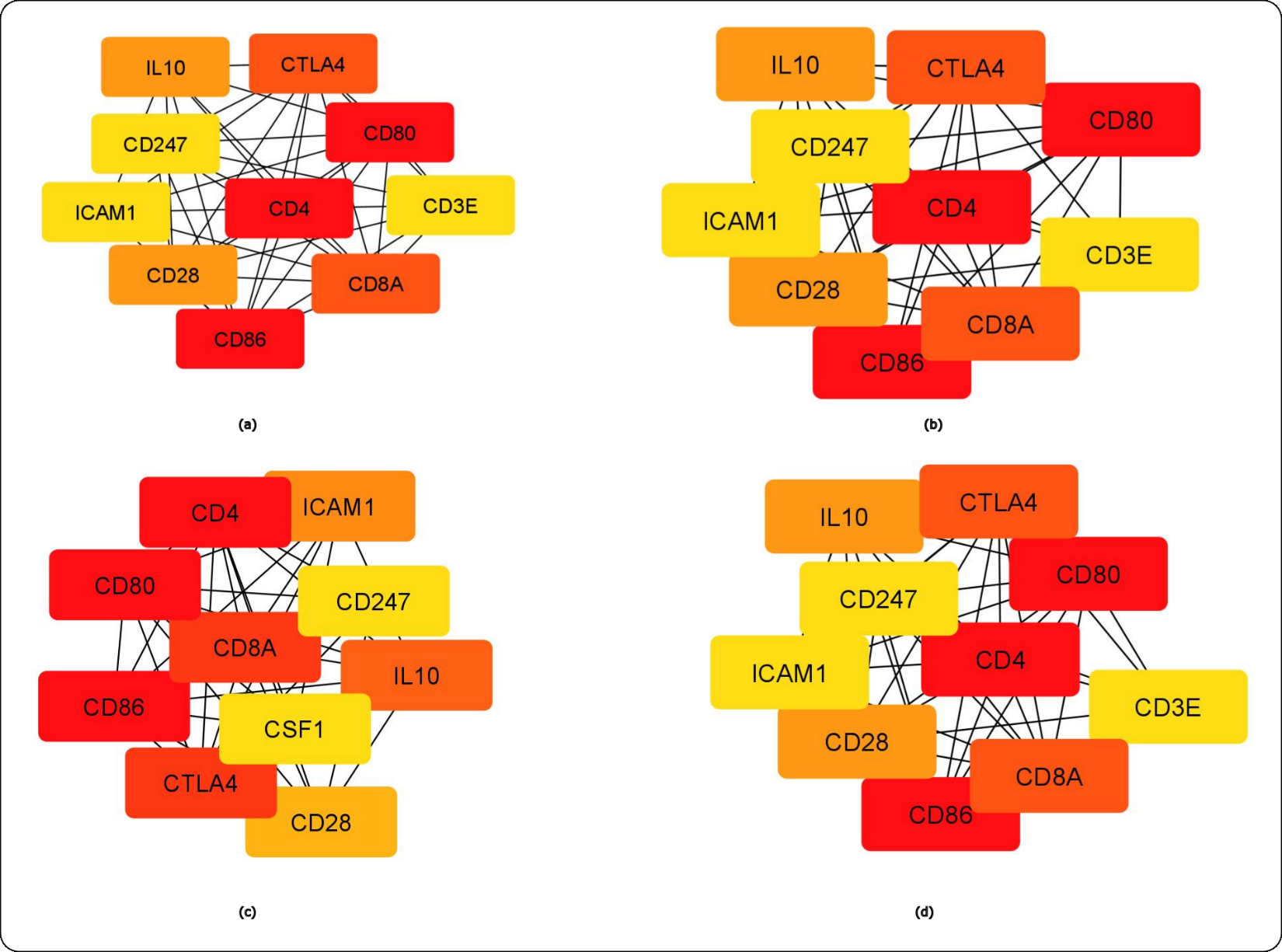


**Supplementary Figure 7: Top-ranking genes of shared common gene CD86 shared between datasets co-expression network using cytoHubba. (A)** Degree, **(B)** Closeness**, (C)** Betweenness and **(D)** MCC.


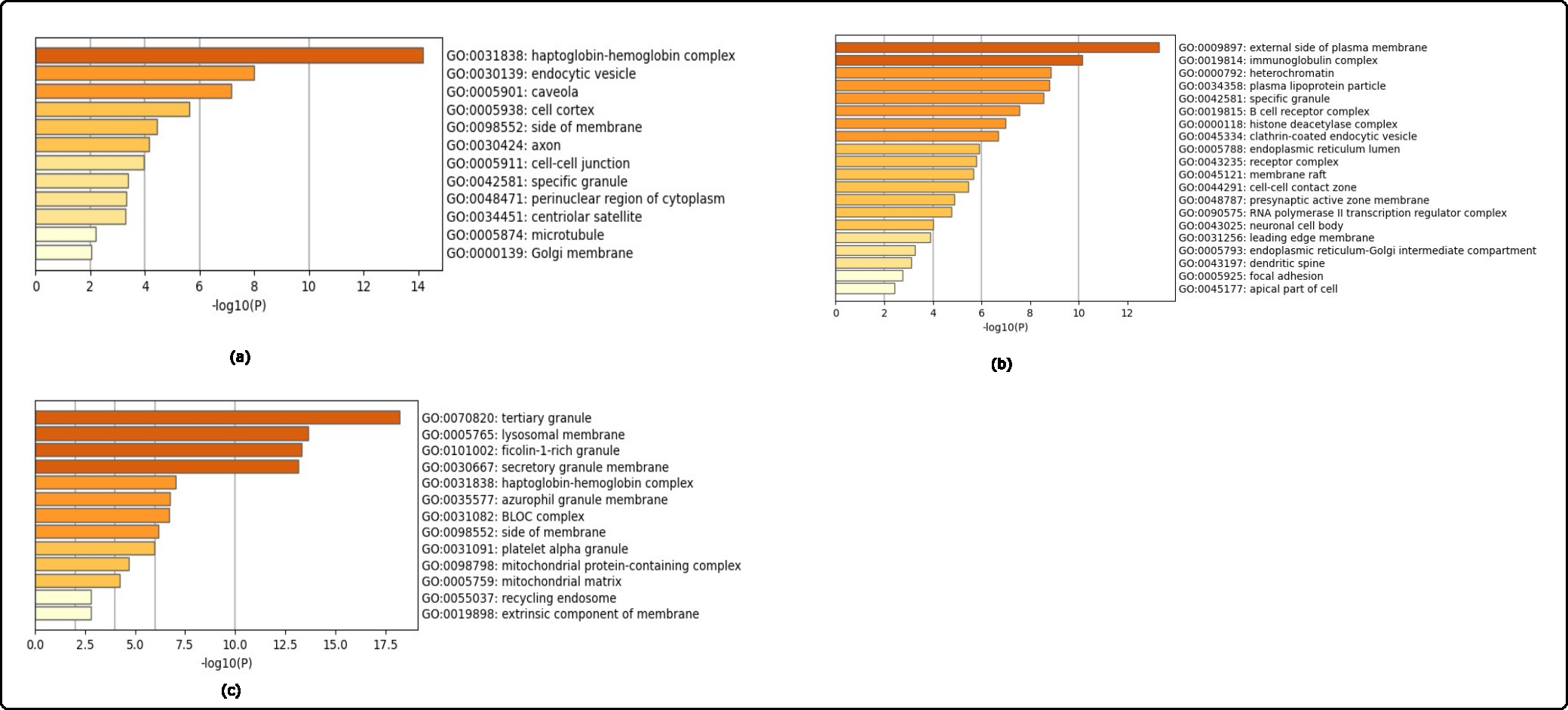


**Supplementary Figure 8: GO-CC terms for the PD and MDD datasets.** **(A)** GO-CC terms for GSE49126 dataset **(B)** GO-CC terms for GSE72267 dataset and **(C)** GO-CC terms for GSE39653 dataset.


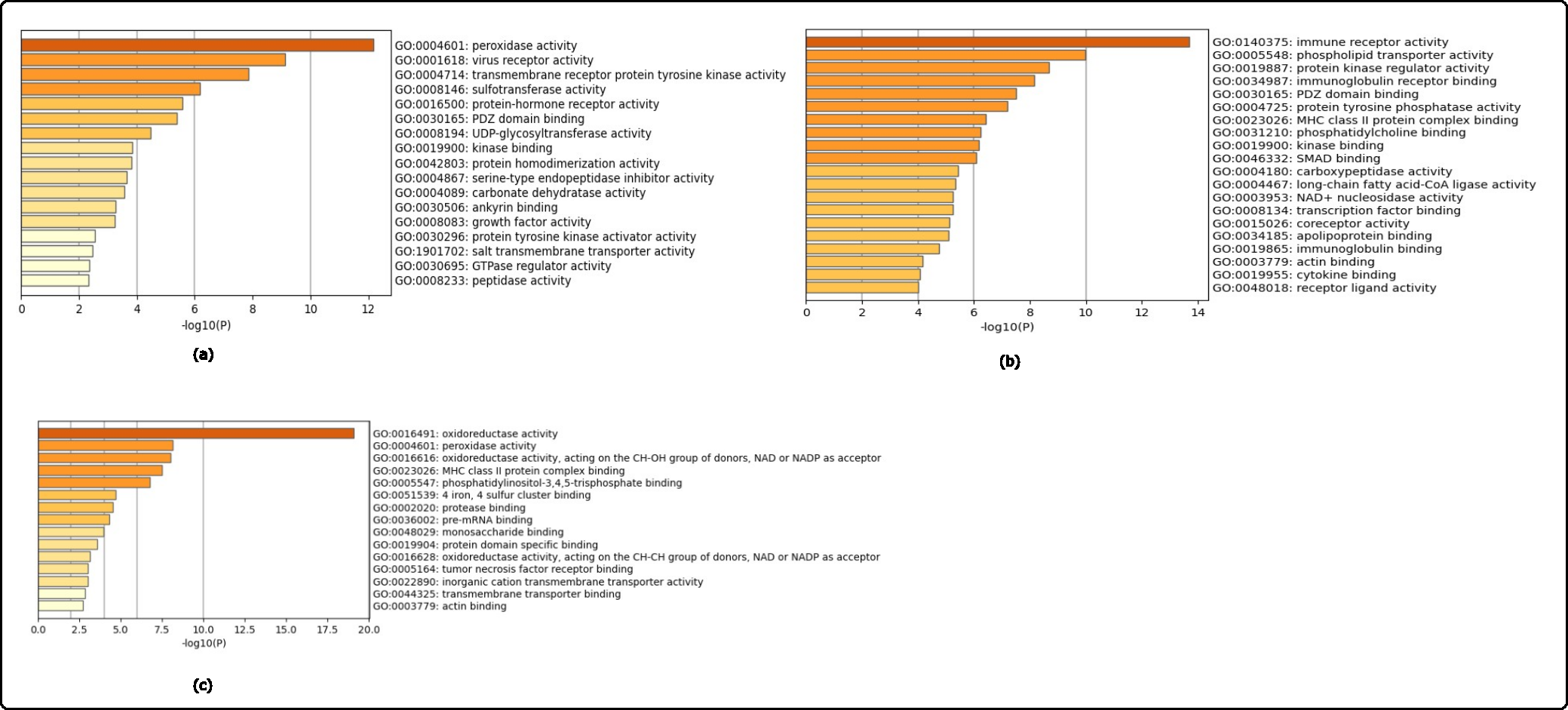


**Supplementary Figure 9: GO-MF terms for the PD and MDD datasets**. **(A)** GO-MF terms for GSE49126 dataset **(B)** GO-MF terms for GSE72267 dataset and **(C)** GO-MF terms for GSE39653 dataset.


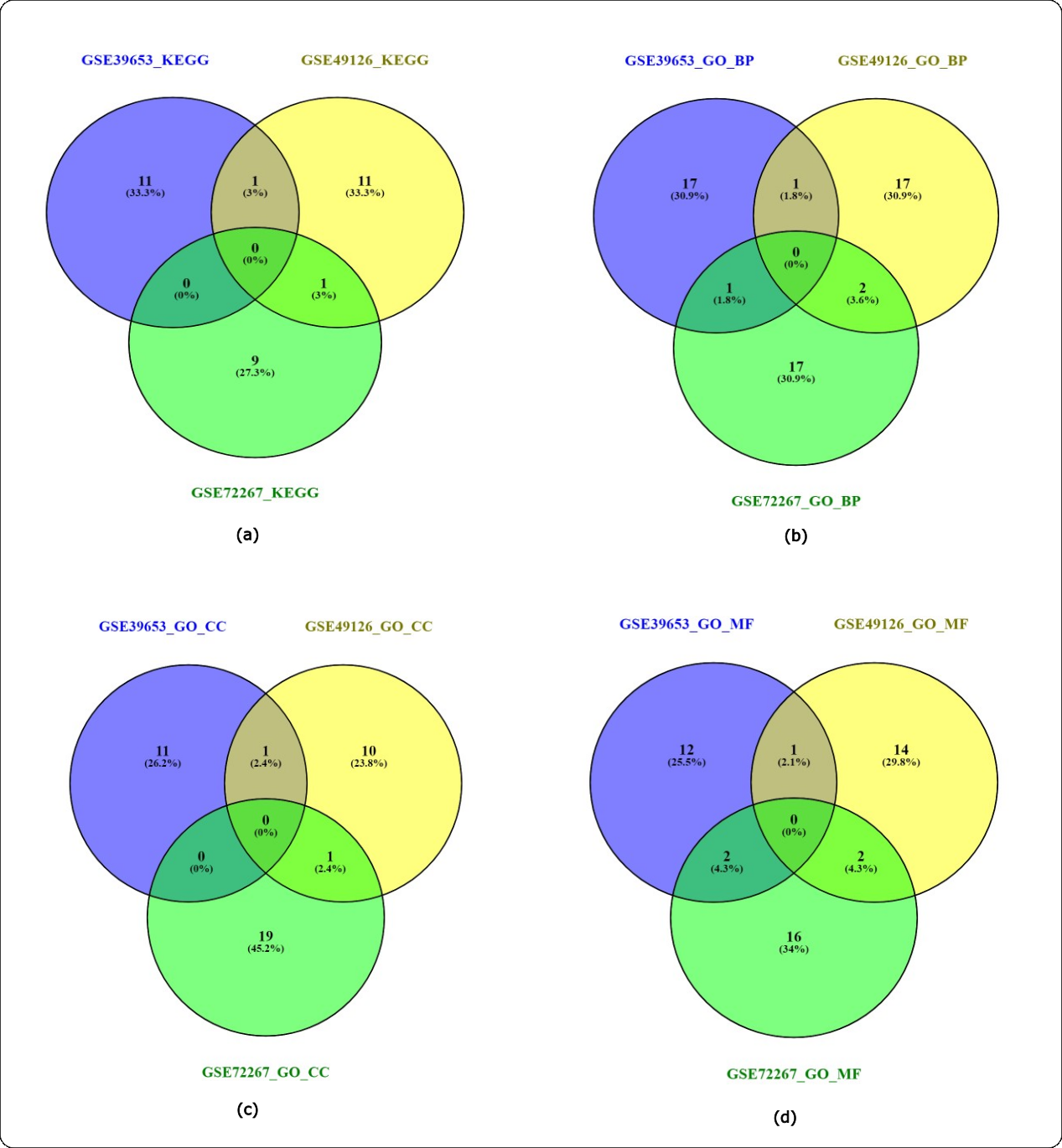


**Supplementary Figure 10: Venn diagram of common and exclusive KEGG pathways and GO terms between the GSE49126, GSE72267 and GSE39653 datasets.** **(A)** Common and exclusive KEGG pathways across the three datasets. **(B)** Common and exclusive GO-BP terms across the three datasets. **(C)** Common and exclusive GO-CC terms across the three datasets. **(D)** Common and exclusive GO-MF terms across the three datasets.

**Supplementary Table 1:** **Number of common and exclusive KEGG pathways shared between the GSE49126, GSE72267 and GSE39653 datasets**

| **Shared common and exclusive KEGG pathways between the three datasets** |
| --- |
| **11 exclusive KEGG pathways GSE39653** |
| NF-kappa B signaling pathway |
| Glutathione metabolism |
| Osteoclast differentiation |
| African trypanosomiasis |
| Herpes simplex virus 1 infection |
| Chemical carcinogenesis - reactive oxygen species |
| Thyroid cancer |
| Ferroptosis |
| Homologous recombination |
| Central carbon metabolism in cancer |
| Spliceosome |
|  |
| **11 exclusive KEGG pathways GSE49126** |
| Malaria |
| Complement and coagulation cascades |
| Bile secretion |
| Longevity regulating pathway - multiple species |
| Pathways in cancer |
| Glycosaminoglycan biosynthesis - heparan sulfate / heparin |
| Rheumatoid arthritis |
| Endocytosis |
| Calcium signaling pathway |
| Nitrogen metabolism |
| Hippo signaling pathway |
|  |
| **9 exclusive KEGG pathways GSE72267** |
| B cell receptor signaling pathway |
| Cytokine-cytokine receptor interaction |
| TGF-beta signaling pathway |
| Fatty acid metabolism |
| Insulin secretion |
| Cholesterol metabolism |
| TNF signaling pathway |
| Thyroid hormone signaling pathway |
| Salmonella infection |
|  |
| **1 common KEGG pathways GSE39653 & GSE49126** |
| Intestinal immune network for IgA production |
|  |
| **1 common KEGG pathways GSE49126 & GSE72267** |
| Hematopoietic cell lineage |

**Supplementary Table 2:** **Number of common and exclusive GO-BP terms shared between the GSE49126, GSE72267 and GSE39653 datasets**

| **Shared common and exclusive GO-BP terms between the three datasets** |
| --- |
| **17 exclusive GO-BP terms GSE39653** |
| positive regulation of leukocyte activation |
| regulation of innate immune response |
| adaptive immune response based on somatic recombination of immune receptors built from immunoglobulin superfamily domains |
| regulation of reactive oxygen species metabolic process |
| cellular response to toxic substance |
| response to wounding |
| response to nutrient levels |
| generation of precursor metabolites and energy |
| hormone metabolic process |
| activation of protein kinase C activity |
| monocarboxylic acid metabolic process |
| regeneration |
| hexose metabolic process |
| response to alcohol |
| cellular response to chemical stress |
| endocytosis |
| mitochondrion organization |
|  |
| **17 exclusive GO-BP terms GSE49126** |
| tube morphogenesis |
| regulation of leukocyte activation |
| oxygen transport |
| gland development |
| regulation of vesicle-mediated transport |
| immune effector process |
| positive regulation of response to external stimulus |
| regulation of protein polymerization |
| placenta development |
| rhythmic process |
| negative regulation of oxidoreductase activity |
| nucleotide catabolic process |
| regulation of epithelial cell proliferation |
| regulation of nitric oxide mediated signal transduction |
| response to progesterone |
| exocrine system development |
| regulation of synaptic vesicle cycle |
|  |
| **17 exclusive GO-BP terms GSE72267** |
| regulation of protein kinase activity |
| positive regulation of defense response |
| cell activation |
| regulation of cell activation |
| response to bacterium |
| negative regulation of immune system process |
| response to hormone |
| regulation of immune effector process |
| glycerolipid metabolic process |
| very long-chain fatty acid metabolic process |
| negative regulation of phosphorylation |
| positive regulation of lipid localization |
| muscle tissue development |
| regulation of ERBB signaling pathway |
| negative regulation of locomotion |
| regulation of lipid metabolic process |
| regulation of plasma lipoprotein particle levels |
|  |
| **1 common GO-BP terms GSE39653 & GSE49126** |
| positive regulation of cell death |
|  |
| **2 common GO-BP terms GSE49126 & GSE72267** |
| positive regulation of immune response |
| enzyme-linked receptor protein signaling pathway |
|  |
| **1 common GO-BP terms GSE39653 & GSE72267** |
| cellular response to cytokine stimulus |

**Supplementary Table 3:** **Number of common and exclusive GO-CC terms shared between the GSE49126, GSE72267 and GSE39653 datasets**

| **Shared common and exclusive GO-CC terms between the three datasets** |
| --- |
| **11 exclusive GO-CC terms GSE39653** |
| tertiary granule |
| lysosomal membrane |
| ficolin-1-rich granule |
| secretory granule membrane |
| azurophil granule membrane |
| BLOC complex |
| platelet alpha granule |
| mitochondrial protein-containing complex |
| mitochondrial matrix |
| recycling endosome |
| extrinsic component of membrane |
|  |
| **10 exclusive GO-CC terms GSE49126** |
| endocytic vesicle |
| caveola |
| cell cortex |
| side of membrane |
| axon |
| cell-cell junction |
| perinuclear region of cytoplasm |
| centriolar satellite |
| microtubule |
| Golgi membrane |
|  |
| **19 exclusive GO-CC terms GSE72267** |
| external side of plasma membrane |
| immunoglobulin complex |
| heterochromatin |
| plasma lipoprotein particle |
| B cell receptor complex |
| histone deacetylase complex |
| clathrin-coated endocytic vesicle |
| endoplasmic reticulum lumen |
| receptor complex |
| membrane raft |
| cell-cell contact zone |
| presynaptic active zone membrane |
| RNA polymerase II transcription regulator complex |
| neuronal cell body |
| leading edge membrane |
| endoplasmic reticulum-Golgi intermediate compartment |
| dendritic spine |
| focal adhesion |
| apical part of cell |
|  |
| **1 common GO-CC GSE39653 & GSE49126** |
| haptoglobin-hemoglobin complex |
|  |
| **1 common KEGG pathways GSE49126 & GSE72267** |
| specific granule |

**Supplementary Table 4:** **Number of common and exclusive GO-MF terms shared between the GSE49126, GSE72267 and GSE39653 datasets**

| **Shared common and exclusive GO-MF terms between the three datasets** |
| --- |
| **12 exclusive GO-MF terms GSE39653** |
| oxidoreductase activity |
| oxidoreductase activity, acting on the CH-OH group of donors, NAD or NADP as acceptor |
| phosphatidylinositol-3,4,5-trisphosphate binding |
| 4 iron, 4 sulfur cluster binding |
| protease binding |
| pre-mRNA binding |
| monosaccharide binding |
| protein domain specific binding |
| oxidoreductase activity, acting on the CH-CH group of donors, NAD or NADP as acceptor |
| tumor necrosis factor receptor binding |
| inorganic cation transmembrane transporter activity |
| transmembrane transporter binding |
|  |
| **12 exclusive GO-MF terms GSE49126** |
| virus receptor activity |
| transmembrane receptor protein tyrosine kinase activity |
| sulfotransferase activity |
| protein-hormone receptor activity |
| UDP-glycosyltransferase activity |
| protein homodimerization activity |
| serine-type endopeptidase inhibitor activity |
| carbonate dehydratase activity |
| ankyrin binding |
| growth factor activity |
| protein tyrosine kinase activator activity |
| salt transmembrane transporter activity |
| GTPase regulator activity |
| peptidase activity |
|  |
| **16 exclusive GO-MF terms GSE72267** |
| immune receptor activity |
| phospholipid transporter activity |
| protein kinase regulator activity |
| immunoglobulin receptor binding |
| protein tyrosine phosphatase activity |
| phosphatidylcholine binding |
| SMAD binding |
| carboxypeptidase activity |
| long-chain fatty acid-CoA ligase activity |
| NAD+ nucleosidase activity |
| transcription factor binding |
| coreceptor activity |
| apolipoprotein binding |
| immunoglobulin binding |
| cytokine binding |
| receptor ligand activity |
| **1 common GO-MF terms GSE39653 & GSE49126** |
| peroxidase activity |
|  |
| **2 common GO-MF terms GSE49126 & GSE72267** |
| PDZ domain binding |
| kinase binding |
|  |
| **1 common GO-MF terms GSE39653 & GSE72267** |
| MHC class II protein complex binding |
| actin binding |
